# Prevalence and genetic characterization of clinically relevant extended-spectrum β-lactamase-producing *Enterobacterales* in the Gulf Cooperation Council countries

**DOI:** 10.3389/frabi.2023.1177954

**Published:** 2023-06-26

**Authors:** Hamad A. Hadi, Hissa Al-Hail, Leena Elsheikh Aboidris, Mahmood Al-Orphaly, Mazen A. Sid Ahmed, Bincy Gladson Samuel, Hana Adam Mohamed, Ali A. Sultan, Sini Skariah

**Affiliations:** ^1^Department of Infectious Diseases, Communicable Diseases Centre, Hamad Medical Corporation, Doha, Qatar; ^2^Department of Medical Education, Weill Cornell Medicine – Qatar, Education City, Qatar Foundation, Doha, Qatar; ^3^Laboratory Services, Philadelphia Department of Public Health, Philadelphia, PA, United States; ^4^The Life Science Centre, School of Science and Technology, Örebro University, Örebro, Sweden; ^5^Department of Microbiology and Immunology, Weill Cornell Medicine – Qatar, Education City, Qatar Foundation, Doha, Qatar

**Keywords:** *Enterobacterales*, antibiotic resistance, multidrug resistance, Gulf Cooperation Council, *E. coli*, *K. pneumoniae*

## Abstract

**Introduction:**

Among Gram-negative bacteria (GNB), *Enterobacterales* (*Enterobacterales*), such as *Escherichia coli* (*E. coli*) and *Klebsiella pneumoniae* (*K. pneumoniae*), are the most clinically relevant pathogens in healthcare settings. Infections secondary to these pathogens are widely common but multidrug resistance (MDR) in *Enterobacterales* has become a significant challenge with increased morbidity, mortality, and cost of management. The escalating global prevalence of MDR in *Enterobacterales* has led to limited treatment options, raising an urgent need for novel antimicrobial therapy(s) and detailed studies exploring underlying resistance mechanisms. In *Enterobacterales*, the prime antimicrobial resistance mechanism against β-lactam antibiotics is mainly the production of β-lactamases, particularly extended-spectrum β-lactamases (ESBLs). Although the Gulf region is witnessing major challenges from infections secondary to MDR GNB, the extent of the problem has not been fully evaluated. Therefore, this review aims to address the prevalence and genetic characterization of ESBL-producing *Enterobacterales* in the Gulf Cooperation Council (GCC) countries.

**Methods:**

PubMed® (National Library of Medicine, Bethesda, MD, USA) search was conducted, which looked for academic articles discussing the epidemiology of MDR *Enterobacterales* in the GCC countries, published in the last 5 years.

**Results and conclusions:**

In GCC countries there is a high prevalence rate of MDR *Enterobacterales*, particularly ESBLs. Prevalence rates of ESBL-producing *Enterobacterales* among the *Enterobacterales* in general clinical samples in the GCC region is 21.6%–29.3%, with a slightly higher prevalence rate in intensive care unit patients (17.3–31.3%) and in patients with urinary tract infections (25.2%–31.7%). ESBL carriers have also been noted in the general community. ESBL-producing *Enterobacterales* from the GCC region show high levels of resistance to ampicillin, aztreonam, third-/fourth-generation cephalosporins, fluoroquinolones, and trimethoprim-sulfamethoxazole. Intermediate resistance rates are observed against nitrofurantoin, piperacillin/tazobactam, and gentamicin, with increasing resistance observed against tigecycline. The isolates demonstrate low-level resistance to carbapenems, fosfomycin, colistin, and amikacin. *Enterobacterales* isolates that are concomitant ESBL producers and are carbapenem resistant have been increasingly reported and demonstrate alarmingly increased antibiotic resistance patterns compared with ESBL *Enterobacterales*. The most prevalent genes for ESBL resistance in the *Enterobacterales* isolates in the GCC region are: *bla*_CTX-M_ (subtype group 1) followed by/co-dominated by *bla*_TEM_ and *bla*_SHV_, whereas the most common carbapenem-resistant genes are *bla*_OXA-48_ and *bla*_NDM-1_.

## 1 Introduction

Over recent decades, antimicrobial resistance (AMR) has risen as a global concern because of substantial increases in morbidity and mortality, and because of the associated costs of management (Bassetti et al., 2017; Naylor et al., 2018). At its forefront are Gram-negative bacteria, (GNB) such as *Enterobacterales* (*Enterobacterales*), *Pseudomonas aeruginosa*, and *Acinetobacter baumannii*, which have become increasingly resistant to most conventional and broad-spectrum antimicrobial agents, including carbapenems (Nordmann and Poirel, 2019). A subset of the GNB family, the order of *Enterobacterales* consists of Gram-negative bacilli that predominantly colonize the human gastro-intestinal tract. In healthcare settings, the clinically important pathogens encompass *Escherichia coli*, *Klebsiella* species, *Enterobacter* species, *Proteus* species*, Citrobacter* species, *Salmonella* species*, Shigella* species, and *Serratia marcescens.* Following the discovery of antibiotics in the last century, these bacteria have accumulated various AMR mechanisms, enabling them to become multidrug resistant, resulting in a noticeable increase in morbidity and mortality (Exner et al., 2017). Owing to their capacity to disseminate as healthcare-associated infections (HCAIs) together with the rapid acquisition of diverse mechanisms of resistance, the World Health Organization has listed cephalosporin- and carbapenem-resistant *Enterobacterales* as multidrug-resistant priority pathogens in need of immediate advances in research and development and newer therapeutic options (WHO, 2017).

### 1.1 Mechanisms of resistance in *Enterobacterales*

According to an agreed international consensus, multidrug-resistant GNB refer to pathogens that are non-susceptible to at least one agent from three different antimicrobial classes, where extensively drug-resistant (XDR) bacteria are susceptible to only two or less antimicrobial classes, and pandrug-resistant (PDR) bacteria are not susceptible to all routinely tested antimicrobials (Exner et al., 2017). This is widely evident in *Enterobacterales*, which express resistance to several classes, including penicillins, cephalosporins, aminoglycosides, quinolones, and sulfonamides, as well as broad-spectrum agents, such as tigecycline, carbapenems, and polymyxins. This resistance can be gained either intrinsically via chromosomal inheritance/through *de novo* mutations or, more commonly, extrinsically via plasmid-mediated mobile horizontal gene transfer (Ruppe et al., 2015) (Partridge, 2015). Resistance genes can be transferred between chromosomes and plasmids via mobile genetic elements, such as insertion sequences, integron systems, or transposons (Partridge, 2015).

One of the key resistance mechanisms in *Enterobacterales* is the production of β-lactamases, particularly extended-spectrum β-lactamases (ESBLs), which hydrolyze β-lactam-based antibiotics such as penicillins, cephalosporins, and monobactams (Bush, 2018). As per the pivotal Ambler classification system, β-lactamases are categorized into classes A to D based on their amino acid sequences (Ruppe et al., 2015). Although classes A, C, and D are serine-based proteases, class B are zinc-based metallo-β-lactamases (MBLs) (Walther-Rasmussen and Høiby, 2007). Class A β-lactamases include penicillinases (TEM, SHV), ESBLs (TEM, SHV, and CTX-M), and carbapenemases (KPC, GES, IMI, SME, SFC, and NMC-A) [7, 9]. Historically, genomic studies on class A β-lactamases were the first to be described, which include penicillinases encoded by *bla*_TEM_, *bla*_SHV_, and *bla*_CTX-M_. Penicillinases are named as such because of their hydrolyzing effect on benzylpenicillin and ampicillin (Walther-Rasmussen and Høiby, 2007). After the introduction of third-generation cephalosporins, certain penicillinases acquired ESBL phenotype via amino acid substitutions in their active sites (Partridge, 2015). Hence, ESBL-producing GNB are resistant to most β-lactams except for a subset of drugs, including carbapenems, which became the *sine qua non* for the management of ESBL infections, particularly for invasive disease (Ruppe et al., 2015). Despite ESBLs conferring resistance to penicillins, third-generation cephalosporins, and monobactams, they remain vulnerable to β-lactamase inhibitors such as clavulanic acid, tazobactam, and sulbactam, which are commonly used in β-lactam–β-lactamase inhibitor therapies (BLBLIs), albeit with raised concerns regarding using these agents for serious invasive diseases (Samarasinghe, 2019; Burillo and Bouza, 2022).

Exploring β-lactamases and other resistance mechanisms expressed by antimicrobial- and multidrug-resistant pathogens highlights the genetic and environmental components of resistance. Accumulated resistance can be either acquired from mobile elements from the environment or from continuous antibiotic exposure, which results in advantageous *de novo* mutations that potentiate the survival and evolution of resistant pathogens (Huttner et al., 2013; Goodman et al., 2016). Factors that drive AMR are increased use of antimicrobials in agriculture and in the animal industry, including the intermediate environment; host factors, such as comorbidities/immune status; healthcare-associated aspects, such as poor adherence to infection control/prevention practices; and inappropriate and excessive antibiotics exposure (Aslam et al., 2021). Furthermore, AMR is further aggravated by the propagation of resistant pathogens facilitated by international travel and population diversity. This context is best highlighted in the Gulf Cooperation Council (GCC) region, which hosts a diverse expatriate population and is at a crossroads for frequent international travel. As an example, in Qatar, previous studies demonstrate significant rates of community ESBL urinary tract infections (UTIs) where immigrant food handlers were carriers for the resistant pathogens (Eltai et al., 2018a; Eltai et al., 2018c; Andres et al., 2020).

### 1.2 Antimicrobial resistance in the Gulf Cooperation Council region

The problem of antibiotic resistance in Gram-negative pathogens is rising globally and in the GCC region. In a recent study of isolates from Saudi Arabia, 90.1% of the tested Gram-negative isolates were multidrug resistant, with 2.7% being XDR, which is comparable to similarly high MDR rates of 74.5% and 97.5% and XDR rates of 1.5% and 7.7% in Egypt and Sudan, respectively, with the ESBL gene *bla*_CTX-M_ as the predominant gene (100%) (Azab et al., 2021). Among *Enterobacterales*, another study from Saudi Arabia identified 81%, 18.2%, and 2.8% of *Enterobacterales* as MDR, XDR, and PDR, respectively (Bandy and Tantry, 2021). Similar to global trends, increased multidrug resistance (MDR) rates in the GCC region have also led to higher morbidity, mortality, and economic costs. For example, studies show that *Klebsiella pneumoniae* and MDR *Enterobacterales* isolates are linked to 42% and 84% of intensive care unit (ICU) mortalities, respectively, in Saudi Arabia, and any increase in MDR rates will further exacerbate the problem (Al Bshabshe et al., 2020; Alkofide et al., 2020). In the United Arab Emirates, it was noted that hospitalized patients with ESBL UTIs have longer hospital stay durations and are treated with a larger number of antibiotics than their counterparts with non-ESBL infections (Ranjan Dash et al., 2018). MDR infections are also at the forefront of medical concerns in the wake of the global COVID-19 pandemic. COVID-19, especially in severe cases, is frequently associated with excessive antimicrobial prescribing and increased observed AMR (Beovic et al., 2020; Abu-Rub et al., 2021; Baiou et al., 2021; Langford et al., 2022), evidently leading a viscous cycle of increased rates of multidrug-resistant bacteria, along with the development of potentially new XDR and PDR strains. In a recent study in Qatar on critically ill COVID-19 patients, an incidence rate of 4.5 per 1,000 ICU days was identified for MDR Gram-negative bacterial infections. MDR *K. pneumoniae* (23.5%) and *E. coli* (12%) were among the major bacteria identified in this cohort, with an ESBL rate of 82.6% and 91.7%, respectively (Baiou et al., 2021).

Although there have been multiple studies that have evaluated the problem of AMR in the region from different perspectives, this comprehensive review of data published in the last 5 years aims to produce an updated report on the prevalence and genomic epidemiology of ESBL-producing *Enterobacterales* in the GCC region.

## 2 Methods, data collection, and analysis

Available publications pertaining to the prevalence of MDR *Enterobacterales* in the Middle East were searched in PubMed® (National Library of Medicine, Bethesda, MD, USA) (Moher et al., 2010). The following Boolean search terms were used for the search: “Specified country” AND (“Enterobacteriaceae” OR “E. coli” OR “Escherichia coli” OR “Klebsiella pneumoniae” OR “K. pneumoniae”) AND (“resistant” OR “resistance “OR “multidrug resistant”). A similar search with the term ESBL was also carried out. Relevant papers that were published within the last 5 years were included with no age restrictions. The following six countries were included in this paper: Bahrain, Kuwait, Qatar, Oman, Saudi Arabia, and United Arab Emirates. When multiple publications were present, the most recent data/publications that covered the objectives were included. For purposeful inclusion in this study, ESBL *Enterobacterales* are defined as *Enterobacterales* that are resistant to β-lactam antibiotics, such as penicillins, oxyimino-cephalosporins (e.g., ceftriaxone, ceftazidime, cefepime, and cefotaxime), and monobactams, as confirmed and reported by routine internal microbiological susceptibility testing (Burillo and Bouza, 2022). Mixed source indicates all clinical specimens, such as lower respiratory tract infections, blood, urine, wound infections, and others.

## 3 Data and discussion

**Figure 1:**
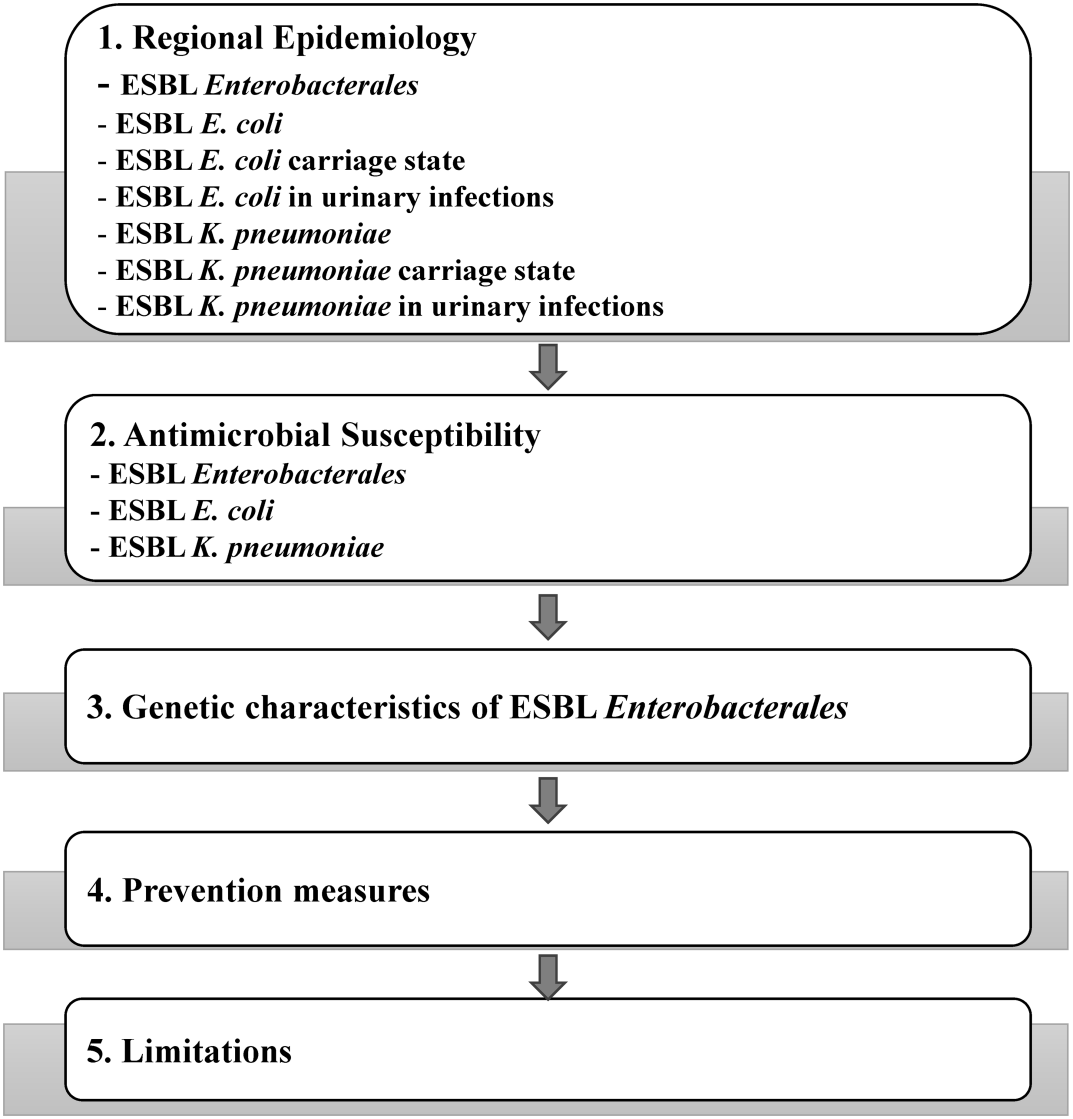
Schematic outline of the date discussed in this review.

The schematic of the data being discussed in this review is shown in **Figure 1**.

### 3.1 Regional epidemiology

#### 3.1.1 ESBL *Enterobacterales*

Overall, the prevalence rate of ESBL-*Enterobacterales* among *Enterobacterales* in general clinical mixed samples was 21.6%–29.3%, with a higher prevalence rate of 17.3%–31.3% in ICU patients and 25.2%–31.7% in patients with UTIs in the GCC region (**Table 1**). The country-specific prevalence rates of ESBL *Enterobacterales* and associated antimicrobial susceptibility data, where applicable, are detailed in **Table 1**.

**Table 1:** ESBL *Enterobacterales* prevalence rate in the GCC region and antimicrobial resistance profile.

Country	Organism	Number of resistant isolates	Source	AMP (%)	AMX/CLV (%)	PIP/TAZ (%)	CLT (%)	CX (%)	CFT (%)	CFZO (%)	CTX (%)	CFZ (%)	CFP (%)	ERP (%)	MNM (%)	IMP (%)	NF (%)	GEN (%)	AMK (%)	TMPS (%)	FQ (%)	AZT (%)	COL (%)	FOS (%)	TG (%)	Reference(s)
Kuwait	ESBL *Enterobacterales*	24% among *Enterobacterales* from hospital-acquired infections among neurocritical care unit patients	Mixed clinical specimens from neurocritical care units	–	–	–	–	–	–	–	–	–	–	–	–	–	–	–	–	–	–	–	–	–	–	Abulhasan et al., 2020
		29.3% among *Enterobacterales*	Mixed (pediatric)	–	33.8	14.6	–	–	–	–	–	75.5	66.2	–	1.3	0.7	–	–	2	–	27.8	83.4	2.2	–	0	Al-Sweih et al., 2021
		13.2% of *Enterobacterales* isolates from food handlers	Fecal samples from food handlers	–	–	–	–	–	–	–	–	–	–	–	–	–	–	–	–	–	–	–	–	–	–	Moghnia et al., 2021
Qatar	ESBL *Enterobacterales*	31.7% among *Enterobacterales*	Urine (pediatric inpatients)	100	99.1	10	100	8.2	**–**	100	100	**–**	97.3	0	0	0	9.1	24.5	0	60.9	36.4	–	–	–	–	Eltai et al., 2018b
		17.3% among *Enterobacterales*	Mixed clinical specimens from ICU	–	99.1	22	–	–	–	–	99.1	–	93.6	2.8	0	0.9	–	33	2.8	61.5	39.4	–	–	–	35.8	Sid Ahmed et al., 2016
		25.2% among UTI patients	Urine	–	–	–	–	–	–	–	–	–	–	–	–	–	–	–	–	–	–	–	–	–	–	Naushad et al., 2022
Saudi Arabia	ESBL *Enterobacterales*	24% of *Enterobacterales*	Mixed	–	–	–	–	–	–	–	–	–	–	5.1	1.3	4.7	–	36.5	13	66.1	50.8–58.6	95.4	–	–	17.8	Aldrazi et al., 2020; Bandy and Tantry, 2021; Ibrahim et al., 2021
		31.3% of *Enterobacterales*	Mixed samples from ICU	–	–	–	–	–	–	–	–	–	–	–	–	–	–	–	–	–	–	–	–	–	–	Alkofide et al., 2020
		80.6% among CRE *Enterobacterales* from inpatients	Mixed CRE isolates	–	–	100	–	84	100	–	–	96	96	44	20	40	–	68	12	–	92	92	4	0	0	Al-Agamy et al., 2018
UAE	ESBL *Enterobacterales*	21.6% among *Enterobacterales*	Mixed clinical specimens	97.8	6.7	2.2	–	–	97.8	–	–	97.8	97.8	0	0	0	2.2	15.6	0	57.8	40–53.3	–	–	0	–	Alfaresi et al., 2018
		–	Urine samples from UTI patients from community	100	–	–	–	–	–	–	–	–	–	–	0	–	–	–	–	73	74	–	–	–	–	Ranjan Dash et al., 2018

Mixed source indicates all clinical specimens, including lower respiratory tract infections, blood, urine, wound infections, etc.

AMK, amikacin; AMP, ampicillin; AMX/CLV, amoxicillin/clavulanate; AZT, aztreonam; CFP, cefepime; CFT, cefotaxime; CFZ, ceftazidime; CFZO, cefazolin; CLT, cephalothin; COL, colistin; CRE, carbapenem resistant; CTX, ceftriaxone; CX, cefoxitin; ERP, ertapenem; FOS, fosfomycin; FQ, fluoroquinolone (ciprofloxacin/norfloxacin/levofloxacin); GEN, gentamicin; IMP, imipenem; MNM, meropenem; NF, nitrofurantoin; PIP/TAZ, piperacillin/tazobactam; TG, tigecycline; TMPS, trimethoprim−sulfamethoxazole; UAE, United Arab Emirates; –, not reported. Ranked by country. % represents resistance rates.

Furthermore, as an indicator of the prevalence of ESBL *Enterobacterales* in the general community, 13.2% of food handlers, who are mainly immigrants, were identified as ESBL *Enterobacterales* carriers in a study conducted in Kuwait in 2021 (Moghnia et al., 2021). When compared with the GCC region, a higher ESBL prevalence has been noted in countries like Thailand (38.4%), Vietnam (55.1%), Cambodia (40.6%), and the Philippines (36.8%) (Suwantarat and Carroll, 2016; Caron et al., 2018; Siriphap et al., 2022).

Globally, among Gram-negative bacterial infections, *E. coli* (33%) is the most isolated organism followed by *K. pneumoniae* (19.2%), hence we decided to focus on these two pathogens exclusively in this review (Karlowsky et al., 2022). As a regional representation, in a longitudinal study over a 6-year period from a large hospital in Saudi Arabia encompassing almost 33,000 GNB isolates, ESBLs were predominated by *E. coli* and *K. pneumoniae* at 20% and 26%, respectively (Al-Tawfiq et al., 2020).

#### 3.1.2 E. coli

In the GCC countries, *E. coli* more commonly demonstrate the non-carbapenem-resistant ESBL phenotype when compared with *K. pneumoniae*, which is similar to some other regions, such as the USA, Canada, and Asia, but in contrast to parts of Europe, where ESBL non-carbapenem-resistant *K. pneumoniae* is more dominant (Karlowsky et al., 2022). Although exceptions to this can also be seen, such as in a previous study from Saudi Arabia (Al-Tawfiq et al., 2020). Overall, ESBL *E. coli* is the dominant organism among ESBL *Enterobacterales* in the GCC region, with a prevalence rate ranging from 34% to 86.6% (**Supplementary Table 1**). Specifically, in case of general mixed samples in the GCC region (excluding ICU patients and urine samples), the prevalence rate of ESBL *E. coli* among *total E. coli* infections has been reported to be 20%–71.3%. **Supplementary Table 1** details country-specific prevalence rates of ESBL *E. coli* and associated antimicrobial susceptibility data, where applicable, which have been ranked according to country and sample type. Kuwait tops the list with the largest number of ESBL non-carbapenem-resistant *E. coli* cases in general clinical samples at 71.3% (**Supplementary Table 1**) (Karlowsky et al., 2022). Among carbapenem-resistant *E. coli*, a study from Saudi Arabia showed a 60% prevalence rate of ESBL *E. coli* among *E. coli* infections (Al-Agamy et al., 2018). Higher prevalence rates were noted in ICU patients, especially among infants, at 79.2% (Almogbel et al., 2021).

Previous reports from Saudi Arabia show that the rates of MDR isolates in mixed clinical samples, especially ESBL *E. coli* and *K. pneumoniae*, were seen to be increasing in the country from 2010 to 2015 (Farah et al., 2019). Specifically, ESBL *E. coli* rates increased from 32% in 2010 to 41% in 2015 in mixed samples (Farah et al., 2019). However, this increasing trend seems to have reversed, with another study showing a decrease in ESBL *E. coli* prevalence rate from 56% in 2016 to 50% in 2017 (Aldawsari et al., 2020). Recent studies from Saudi Arabia also show a lower prevalence of ESBL *E. coli*, ranging from 31.1% to 36.6% (Ibrahim et al., 2019; Aldrazi et al., 2020; Bandy and Tantry, 2021; Ibrahim et al., 2021).

In comparison to general mixed samples, because of expected pathogen, host, and environmental factors, the prevalence rates of ESBL *E. coli* are higher in most studies analyzing specific samples from critical patients, as seen for respiratory samples [100% among respiratory *E. coli* infections (ICU patients)], endotracheal samples (75% among MDR *E. coli*), and blood samples (52.2% among total bloodstream infection-causing *E. coli*) (Bandy and Almaeen, 2020; Kabrah et al., 2021; Sannathimmappa et al., 2021) (**Supplementary Table 1**).

#### 3.1.3 ESBL *E. coli* carriage state

Among healthy carriers, studies carried out in Qatar and Kuwait identified an ESBL *E. coli* prevalence rate of 9% among commensal *E. coli* and of 18.8% among *E. coli* isolates from food handlers, respectively (Eltai et al., 2018c; Moghnia et al., 2021) (**Supplementary Table 1**). This poses a significant risk factor, as any failures in hand/food hygiene among these food handlers could lead to rapid dissemination of these ESBL/carbapenem-resistant (**Supplementary Table 1**) strains in the community, and, subsequently, increase transfer of resistance genes among prevalent bacteria, with the potential for secondary infections (Moghnia et al., 2021). These data also reinforce the need for strict and active monitoring of food hygiene protocols and consistent testing of relevant staff to prevent related hospital and community spread. The potential spread of these MDR bacteria in hospital environments can be exemplified by the latter study from the GCC region where ESBL *E. coli* isolates from healthy food handlers in Kuwait represented 71.2% of food handlers working in healthcare settings and 28.8% of community food handlers (*p* = 0.001) (Moghnia et al., 2021).

#### 3.1.4 ESBL *E. coli* in urinary infections

For urinary infections, in the GCC region, an overall prevalence rate of 4%–50% was seen for ESBL *E. coli* among urinary infection-causing *E. coli* (**Supplementary Table 1**). A higher ESBL *E. coli* prevalence among uropathogenic *E. coli* was seen in hospitalized patients (23.5%–50%) (Kabrah et al., 2021; Mohammed et al., 2022) when compared with studies looking at both inpatients and outpatients (4–33.5%) (Balkhi et al., 2018; Alzahrani et al., 2020; Bazaid et al., 2021; Abalkhail et al., 2022; Badger-Emeka et al., 2022) or specifically outpatients (11.1% in female outpatients and 23% in patients with UTIs in the community) (Ranjan Dash et al., 2018; Alasmary, 2021) (**Supplementary Table 1**). A study from Saudi Arabia noted a lower ESBL *E. coli* prevalence rate among uropathogens in children (3.23%) and adults (4.82%) than in elderly patients (8.33%) samples from emergency department (Alanazi et al., 2018) (**Supplementary Table 1**). A study from Bahrain, encompassing over 3,000 non-repetitive urinary samples from a large tertiary care hospital, showed the one of the highest ESBL *E. coli* prevalence in the GCC region in relation to urinary infections, with rates of ESBL *E. coli* infections increasing from 37.3% in 2018 to 39% in 2019 (Saeed et al., 2021). Another alarming factor is that the majority of ESBL *E. coli* and carbapenem-resistant *E. coli* cases in Bahrain are hospital acquired (51% and 5.9%, respectively) rather than community acquired (37.6% and 0.5%, respectively) (Saeed et al., 2021); however, in the United Arab Emirates, it was observed that > 75% of the ESBL *E. coli* and *K. pneumoniae* cases were from the community (Dash et al., 2008). Similar to mixed clinical samples, one study from Saudi Arabia noted a marked decline in ESBL uropathogenic *E. coli* cases, decreasing from 100% in 2017 to 4% in 2019, among a cohort of both inpatients and outpatients, including emergency and intensive care patients (Badger-Emeka et al., 2022). The authors attribute this decrease to the adoption of antimicrobial stewardship programmes in some hospitals, in addition to the implementation of stringent regulation for prescription antibiotic dispensing in community pharmacies by the Saudi Arabia Ministry of Health (Badger-Emeka et al., 2022). Another study in Saudi Arabia also noted a similar decrease in ESBL *E. coli* and *K. pneumoniae* among uropathogenic *E. coli* and *K. pneumoniae*, from 23.7% and 40%, respectively, in 2015 to 17.6% and 16.7%, respectively, in 2019 in a cohort composed largely of outpatients (Bazaid et al., 2021). Kuwait showed a variable trend, with a study reporting increasing prevalence of ESBL *E. coli* from approximately 18% in 2017 to 37% in 2019, followed by a decrease to 22% in 2021 (Al Benwan and Jamal, 2022).

#### 3.1.5 ESBL *K. pneumoniae*

ESBL *K. pneumoniae* formed the second most dominant organism among ESBL *Enterobacterales* in the region, with a prevalence rate of 11.1%–52.8% (**Supplementary Table 2**). The ESBL *K. pneumoniae* prevalence rate among *K. pneumoniae* isolates in the GCC region ranged from 13%–52.4% for general mixed clinical samples to 7%–87.5% for samples from ICU patients (**Supplementary Table 2**), with the prevalence increasing to 90.5% among carbapenem-resistant *K. pneumoniae* isolates isolated from inpatients. **Supplementary Table 2** details country-specific prevalence rates of ESBL *K. pneumoniae/*spp. and associated antimicrobial susceptibility data, where applicable, which have been ranked according to country and sample type. Kuwait reported the highest prevalence of ESBL non-carbapenem-resistant *K. pneumoniae* (52.4%) among *K. pneumoniae* isolates in marked contrast to lower values reported by the United Arab Emirates and Qatar (20.8% and 28.2%, respectively) in case of general mixed samples (Karlowsky et al., 2022) (**Supplementary Table 2**). The high prevalence rate of ESBL (non-carbapenem-resistant) *K. pneumoniae* (52.4%) in Kuwait, with an equally alarming high rate of ESBL (non-carbapenem-resistant) *E. coli* (71.3%), is similar to the prevalence rates reported by countries like India, Thailand, Vietnam, and Mexico (Karlowsky et al., 2022). One study from Saudi Arabia noted that the prevalence rate of ESBL *K. pneumoniae* showed a modest increase from 22% in 2016 to 26% in 2017 in the country (Aldawsari et al., 2020), whereas another study, looking at isolates from 2014 to 2018, noted that 57.5%–77.8% of the *K. pneumoniae* isolates in the study were resistant to third- and fourth-generation cephalosporins (Al-Zalabani et al., 2020).

The prevalence of ESBL *K. pneumoniae/*spp. among *Klebsiella* isolates across diverse clinical sample types is similar to the range shown by general mixed samples, with prevalence rates of 10%–100% in blood samples, 5.5% among respiratory *K. pneumoniae* infections (ICU patients), 17.1% among MDR *K. pneumoniae* in endotracheal samples, 30% among emphysematous pyelonephritis-causing *K. pneumoniae*, and 25% and 34.3% among total *K. pneumoniae/*spp. in the case of surgical site and device-associated healthcare infections, respectively (**Supplementary Table 2**) (Balkhy et al., 2020; Bandy and Almaeen, 2020; El-Saed et al., 2020; Kabrah et al., 2021; Sannathimmappa et al., 2021; Bazaid et al., 2022; Robles-Torres et al., 2022).

#### 3.1.6 *K. pneumoniae*carriage state

Among healthy carriers, a study from Kuwait identified a prevalence rate of ESBL *K. pneumoniae* of 4% among *K. pneumoniae* from food handlers (Moghnia et al., 2021), indicative of a lower carrier rate of ESBL *K. pneumoniae* in the general population compared with carrier rates of ESBL *E. coli* of 18.8% among *E. coli* isolates from food handlers (Moghnia et al., 2021) (**Supplementary Tables 1**, **2**).

#### 3.1.7 *K. pneumoniae* in urinary infections

In case of ESBL *K. pneumoniae* urinary infections, multiple published studies are available from Saudi Arabia and one study each from the United Arab Emirates, Oman, Kuwait, Qatar, and Bahrain (**Supplementary Table 2**). The prevalence of ESBL *K. pneumoniae* among *K. pneumoniae* causing urinary infections ranged from 10% to 20% in general/mixed patients (adults) (**Supplementary Table 2**), whereas a higher prevalence rate was noted in the pediatric population, ranging from 29.2% to 39.5% (Bazaid et al., 2022; Mohammed et al., 2022). Similar to reports of ESBL *E. coli*, Kuwait showed a mixed trend, with one study reporting an increasing prevalence of ESBL *K. pneumoniae*, from approximately 33% in 2017 to 50% in 2019, followed by a decrease, to 42%, in 2021 (Al Benwan and Jamal, 2022).

### 3.2 Antimicrobial susceptibility

Beta-lactamase Gram-negative producers in the region have been shown to have significantly higher resistance rates to cephalosporins, amoxicillin/clavulanate, piperacillin/tazobactam, nitrofurantoin, aztreonam, ciprofloxacin, and trimethoprim/sulfamethoxazole than non-β-lactamase producers (Ibrahim et al., 2019).

#### 3.2.1 ESBL *Enterobacterales*

ESBL *Enterobacterales* collectively in the GCC region, in general, showed a > 95% resistance to ampicillin, with high resistance rates to aztreonam (83.4%–95.4%), trimethoprim/sulfamethoxazole (57.8%–73%), and fluoroquinolone (27.8%–74%), as listed in **Table 1**. Intermediate levels of resistance were seen against gentamicin (15.6%–36.5%), piperacillin/tazobactam (2.2%–22%), and nitrofurantoin (2.2%–9.1%). One study each from Kuwait, Qatar, and Saudi Arabia evaluated the resistance rates of the ESBL *Enterobacterales* isolates against tigecycline, and 0%, 35.8%, and 17.8% of the tested isolates were identified as resistant, respectively (**Table 1**). Low resistance rates were seen against carbapenems (0%–5.1%), amikacin (0%–13%), and colistin (2.2%), with absolute sensitivity to fosfomycin (**Table 1**). ESBL and carbapenem-resistant *Enterobacterales* isolates showed comparatively higher resistance rates against piperacillin/tazobactam (100%), cefoxitin (84%), fluoroquinolone (92%), gentamicin (68%), and colistin (4%) compared with ESBL *Enterobacterales* (**Table 1**). These isolates still showed absolute susceptibility to fosfomycin and tigecycline. One study from Qatar identified that ESBL *Enterobacterales* from ICU patients in the region demonstrated high susceptibility (99.1%) against two new antibiotic combinations of ceftazidime/avibactam and ceftolozane/tazobactam, which could be potentially alternative therapeutic options for treatment of such infections (Ahmed et al., 2022).

#### 3.2.2 *ESBL* E. coli

ESBL E. coli isolates in the region (excluding studies primarily dealing with urinary isolates and concomitant ESBL and carbapenem-resistant isolates) showed a similar profile as depicted by the ESBL *Enterobacterales*, with high–medium resistance rates to ampicillin (94.7%–100%), amoxicillin/clavulanate (5.1%–97.3%), aztreonam (79%–100%), trimethoprim/sulfamethoxazole (52.1%–78.9%), and fluoroquinolone (0%–77%), with lower resistance rates against piperacillin/tazobactam (0%–15.4%), carbapenems (0%–20%), nitrofurantoin (0%–31.6%), gentamicin (0%–32%), amikacin (0%–15.8%), colistin (0%–1%), and tigecycline (0%–4.1%), and absolute sensitivity to fosfomycin, as listed in **Supplementary Table 1**. Isolates from Saudi Arabia were particularly noteworthy, with higher resistance rates toward nitrofurantoin (1%–31.6%), amikacin (0%–15.8%), trimethoprim/sulfamethoxazole (56.2%–78.9%), fluoroquinolone (0%–77%), and tigecycline (0–4.1%) compared with data from Kuwait, Bahrain, Oman, and the United Arab Emirates, where the values for these countries ranged from 0% to 9.9% for nitrofurantoin, 0% to 1.9% for amikacin, 52.1% to 66.8% for trimethoprim/sulfamethoxazole, 39.6% to 59% for fluoroquinolone, and with 0% for tigecycline (**Supplementary Table 1**). The isolates from the United Arab Emirates also showed a atypical low resistance rate to amoxicillin clavulanate at 5.1%, compared with higher resistance rates ranging from 9% to 97.3% in Bahrain, Qatar, Kuwait, and Saudi Arabia (**Supplementary Table 1**). ESBL and carbapenem-resistant *E. coli* isolates showed higher resistance rates to piperacillin/tazobactam (100%), fluoroquinolone (100%), and gentamicin (50%) (**Supplementary Table 1**) than ESBL *E. coli* isolates.

ESBL *E. coli* isolates from urinary infections generally showed, overall, a high resistance to most antibiotics, including ampicillin (47.8%–100%), aztreonam (99.2%–100%), amoxicillin/clavulanate (33.3%–100%), trimethoprim/sulfamethoxazole (33.3%–82%), fluoroquinolone (0%–100%), nitrofurantoin (0%–52.2%), gentamicin (13.7%–63.6%), and tigecycline (0%–77.1%) (**Supplementary Table 1**), except for piperacillin/tazobactam, amikacin, and fosfomycin, that had low resistance rates of 3%–27.1%, 0%–7.7%, and 1.2%–3.7%, respectively (**Supplementary Table 1**). Studies from Bahrain, Oman, Saudi Arabia, and the United Arab Emirates investigating ESBL *E. coli* isolates from urinary infections also reported almost absolute sensitivity to carbapenems in the region (**Supplementary Table 1**). Concerningly, a study from Saudi Arabia reported a colistin resistance rate of 58.4% among ESBL *E. coli* in a cohort of patients with UTIs (Abalkhail et al., 2022). In Oman, a decrease in resistance was seen in *E. coli* isolates to cephalosporins, aminoglycosides, amoxicillin-clavulanic acid, cotrimoxazole, piperacillin/tazobactam, nitrofurantoin, and carbapenems from 2013 to 2018, which the authors attribute to increased prescription of fluoroquinolones, thus effectively putting a halt to resistance development toward other drugs (Al Mamari et al., 2022).

#### 3.2.3 *ESBL* K. pneumoniae

ESBL *K. pneumoniae* in the GCC region (excluding studies primarily dealing with urinary isolates) also showed overall high resistance rates to most antibiotics, including ampicillin (50%–100%), aztreonam (88.2%–100%), amoxicillin/clavulanate (20%–89.7%), trimethoprim/sulfamethoxazole (0%–93.3%), nitrofurantoin (0%–81.6%), fluoroquinolone (0%–56.4%), and gentamicin (28.6%–90%) (**Supplementary Table 2**), and intermediate resistance to amikacin (0%–50%) and piperacillin/tazobactam (0%–35.3%) (**Supplementary Table 2**). Lower rates of resistances were seen against carbapenems (0%–14.63%) and colistin (2.7%) (**Supplementary Table 2**). Few studies from the region also reported absolute sensitivity to fosfomycin (Al-Agamy et al., 2018; Alfaresi et al., 2018; Karlowsky et al., 2022) (**Supplementary Table 2**). Although multiple studies from the region reported absolute sensitivity to tigecycline (Al-Agamy et al., 2018; Al Rahmany et al., 2019; Al-Sweih et al., 2021; Bazaid et al., 2022), a few studies from Saudi Arabia reported higher resistance rates, ranging from 8% to 23.4%, for tigecycline (Aldrazi et al., 2020; Jalal et al., 2023). ESBL and carbapenem-resistant *K. pneumonia* isolates showed comparatively higher resistance rates to piperacillin/tazobactam (100%), fluoroquinolone (89.5%), and colistin (5.3%) than ESBL *K. pneumonia (* **Supplementary Table 2**). In a 10-year study (2011–2021), *K. pneumonia* isolates in Saudi Arabia were noted as becoming increasingly resistant to ceftazidime, cefotaxime, and cefepime, with resistance rates of 29.9%, 26.2%, and 53.9%, respectively, in 2011 and resistance rates of 84.9%, 85.1%, and 85.8%, respectively, in 2021 (Jalal et al., 2023).

ESBL *K. pneumoniae* isolates from urinary infections once again showed high rates of resistance to ampicillin (58.3%–100%), amoxicillin/clavulanate (69%–100%), nitrofurantoin (14%–57%), trimethoprim/sulfamethoxazole (14%–85%), fluoroquinolone (0%–48%), gentamicin (0%–83.3%), amikacin (0%–57%), tigecycline (0%–41.7%), and cefoxitin (8.3%–100%) (**Supplementary Table 2**), and showed lower rates of resistance to only piperacillin/tazobactam (7%–17%) and carbapenems (0%–7%) (**Supplementary Table 2**), with absolute sensitivity to colistin, as reported in one study from Saudi Arabia (Alzahrani et al., 2020). In Oman, a decrease in resistance was seen in *K. pneumoniae* isolates to cephalosporins and ciprofloxacin, whereas an increasing resistance was noted toward amikacin, amoxicillin-clavulanic acid, cotrimoxazole, nitrofurantoin, and carbapenems from 2013 to 2018. The authors attribute this to a decline in the number of prescriptions of cephalosporins, which subsequently reduced further resistance development against them (Al Mamari et al., 2022).

Despite the reported alternative options for ESBL infections, such as nitrofurantoin, quinolones, and cotrimoxazole, regional genetic variations must also be taken into consideration. For example, high rates of glucose-6-phosphate dehydrogenase (G6PD) deficiency is prevalent in the region, particularly in the Bahraini population (rates of G6PD deficiency in newborn males and females were 18% and 10%, respectively) (Al-Arayyed et al., 2007; Saeed et al., 2021). These observations preclude the liberal use of drugs such as ciprofloxacin, nitrofurantoin, and trimethoprim-sulfamethoxazole for fear of inducing hemolytic crises in G6PD-deficient recipients (Saeed et al., 2021).

### 3.3 Genetic characteristics

Among the studies from different countries in the GCC region, it was clear that the *bla*_CTX-M_ gene is the dominant ESBL gene among ESBL *Enterobacterales* (39.6%–100%), *E. coli* (55.5%–100%), and *K. pneumoniae* (32.1%–100%) in this region across different sample types and clinical settings (**Table 2**). Specifically, the enzymes belonging to the CTX-M group 1, which include CTX-M1, CTX-M-15, and CTX-M-28 enzymes (Dehshiri et al., 2018), are predominant. The *bla*_CTX-M-15_ gene was reported as being the most prevalent ESBL gene in isolates in the gulf region, and this is still the case in many studies from the region (Alqasim et al., 2018; Bin Thani, 2018; Ibrahim et al., 2021). However, this is being replaced by *bla*_CTX-M-28_ in some countries in the GCC region (e.g., the United Arab Emirates), and is similar to trends being reported in countries like South Korea (Yoo et al., 2010; Alfaresi et al., 2011; Alfaresi et al., 2018). Both genes are extremely similar to each other, with a difference of only one amino acid, and they both belong to the same CTX-M group 1 (Alfaresi et al., 2018). The genes identified in ESBL *Enterobacterales* isolates from the GCC region and specifically in ESBL *E. coli* and *K. pneumoniae* are detailed in **Table 2**. Colistin resistance genes (*mcr-1* and *mcr-9*) have also been reported in *Enterobacterales* isolates from the region (Perez-Lopez et al., 2020; Tsui et al., 2020a; Tsui et al., 2020b).

**Table 2:** Antimicrobial resistance genes prevalent in ESBL *Enterobacterales* from the GCC region.

Country	Organism	Source	Resistance genes	Reference(s)
Qatar	ESBL *Enterobacterales*	Urine (pediatric inpatients)	*bla*_CTX-M-_ (59%) [*bla*_CTX-M-G1_ (89.2%), *bla*_CTX-M-G9_ (7.7%), *bla*_CTX-M-G2_ (0.9%), *bla*_CTX-M-G8_ (1.5%)], *bla*_TEM_ (2.7%), *bla*_SHV_ (0.9%)	Eltai et al., 2018b
		Mixed from ICU	*bla*_CTX-M [mainly CTX-M-1]_ (66.1%), *bla*_TEM_ (40.4%), *bla*_SHV_ (53.2%), *bla*_CTX-M_ + *bla*_TEM_ + *bla*_SHV_ (24.7%), *bla*_TEM_ + *bla*_SHV_ (1.8%), *bla*_CTX-M_ + *bla*_TEM_ (10.1%), *bla*_CTX-M_ + *bla*_SHV_ (10.1%)	Sid Ahmed et al., 2016
Saudi Arabia	ESBL *Enterobacterales*	Mixed	*bla*_TEM_ (56.6%, *bla*_TEM-1_ dominant), *bla*_CTX-M_ (17%, *bla*_CTX-M-15_ dominant), *bla*_OXA-1_ (1.9%), *bla*_CTX-M_ + *bla*_TEM_ (22.6%), *bla*_TEM_ + *bla*_SHV_ (1.9%)	Ibrahim et al., 2021
UAE	ESBL *Enterobacterales*	Mixed	*bla*_CTX-M_ (100%) [*bla*_CTX-M-28_ (82.2%), *bla*_CTX-M-167_ (4.4%), *bla*_CTX-M-38, 163, and 198_ (2.2% each)], *bla*_TEM_ (100%) [*bla*_TEM-171_ (17.8%), *bla*_TEM-206_(4.4%), *bla*_TEM-120 and 163_ (2.2% each)], *bla*_SHV_ (44.4%) [*bla*_SHV-148 and 187_ (2.2% each)] and *bla*_VIM_ (4.4%)	Alfaresi et al., 2018
Bahrain	ESBL EC	Mixed	*bla*_CTX-M-15/14/27_ (100%), *bla*_TEM_ (81.2%), *bla*_SHV_ (43.7%), AmpC phenotype noted in 8.9% (CIT and DHA subtype)	Bin Thani, 2018; Joji et al., 2021
Kuwait	ESBL EC	Mixed (pediatric)	*bla*_CTX-M-15_ (87.1%), *bla*_CTX-M-14_ (4.8%), *bla*_CTX-M-27_ (3.2%), *bla*_CTX-M-3_ (1.6%), *bla*_CTX-M-type_ (1.6%)	Al-Sweih et al., 2021
Qatar	ESBL EC	Mixed from ICU	*bla*_CTX-M [mainly CTX-M-1]_ (76.3%), *bla*_TEM_ (34.2%), *bla*_SHV_ (7.9%), *bla*_CTX-M_ + *bla*_TEM_ + *bla*_SHV_ (0%), *bla*_TEM_ + *bla*_SHV_ (5.3%), *bla*_CTX-M_ + *bla*_TEM_ (18.4%), *bla*_CTX-M_ + *bla*_SHV_ (2.6%)	Sid Ahmed et al., 2016
		Mixed (general pediatrics)	**ESBL genes:** *bla*_CTX-M-15_ (84.5%), *bla*_CTX-M-14_ (4.7%), *bla*_TEM-104_ (3%), *bla*_CTX-M-102_ (3%), *bla*_CTX-M-159_ (3%), *bla*_CTX-M-3_ (1.2%), *bla*_CTX-M-27_ (0.6%), *bla*_CTX-M-1_ (0.6%) **Other β-lactamase genes**: *bla*_TEM-1B_ (35.7%), *bla*_OXA-1_ (13.7%), *bla*_NDM1/5_ (3%), *bla*_DHA_ (3.6%), *bla*_TEM-33_*_/_*_35_ (3.6%), *bla*_OXA-48_ (2.4%), *bla*_CMY_ (1.2%) **Plasmid-mediated quinolone resistance genes**: *aac(6′)-Ib-cr* (12.5%), *qnr A/B/E/S* (38.1%) **Aminoglycoside-modifying enzyme genes**: *aac(3)-II [aac(3)-IIa, aac(3)-IId, aac(3)-IVa*, and *aac(3)-VIa*] (16.7%), *aadA1* (10.7%), *aadA2* (3.6%), *aadA5* (30.4%), *aph(3′)-Ia* (7.7%), *aph(3*^”^*)-Ib* (42.8%), *aph(6)-Id* (41.1%) **Cotrimoxazole resistance genes**: *dfrA/B* (67.9%), *sul1-3* (66.7%)	Perez-Lopez et al., 2020
		Mixed (neonatal ICU)	**ESBL genes:** *bla*_CTX-M-15_ (100%) **Other β lactamase genes**: *bla*_TEM-1B_ (73.7%), *bla*_OXA-1_ (26.3%) **Plasmid-mediated quinolone resistance genes**: *aac(6′)-Ib-cr* (15.8%), *qepA1* (10.5%), *qnr A/B/E/S* (36.8%) **Aminoglycoside-modifying enzyme genes**: *aac(3)-II [aac(3)-IIa, aac(3)-IId, aac(3)-IVa*, and *aac(3)-VIa*] (26.3%), *aadA2* (31.6%), *aadA5* (5.3%), *aph(3*^”^*)-Ib* (31.6%), *aph(6)-Id* (31.6%) **Cotrimoxazole resistance genes**: *dfrA/B* (68.4%), *sul1-3* (73.7%)	Perez-Lopez et al., 2020
		Mixed (pediatric ICU)	**ESBL genes:** *bla*_CTX-M-15_ (83.5%), *bla*_TEM-104_ (4.5%), *bla*_CTX-M-102_ (4.5%), *bla*_CTX-M-159_ (3%), *bla*_CTX-M-3_ (1.5%), *bla*_CTX-M-27_ (3%), *bla*_CTX-M-1_ (3%) **Other β-lactamase genes**: *bla*_TEM-1B_ (35.8%), *bla*_OXA-1_ (10.4%), *bla_NDM_*_1_*_/_*_5_ (3%), *bla*_OXA-48_ (1.5%), *bla*_CMY_ (4.5%) **Plasmid-mediated quinolone resistance genes**: *aac(6′)-Ib-cr* (11.9%), *oqxAB* (1.5%), *qnr A/B/E/S* (41.8%) **Aminoglycoside-modifying enzyme genes**: *aac(3)-II [aac(3)-IIa, aac(3)-IId, aac(3)-IVa*, and *aac(3)-VIa*] *(16.4%), aadA1* (14.9%), *aadA2* (7.7%), *aadA5 (32.3%), aph(3′)-Ia* (3%), *aph(3*^”^*)-Ib* (47.8%), *aph(6)-Id* (49.3%) **Cotrimoxazole resistance genes**: *dfrA/B* (68.7%), *sul1-3* (68.7%)	Perez-Lopez et al., 2020
		Fecal (EPEC + EAEC) (pediatric inpatients with acute gastroenteritis)	*bla*_CTX-M-_ (100%) [*bla*_CTX-M-G1 (3 and15)_ (88.23%), *bla*_CTX-M-G1 (8 and14)_ (11.8%), *bla*_CTX-M-G1 (2 and 9)_ (5.9%)], *bla*_TEM_ (70.6%), *bla*_SHV_ (5.9%)	Eltai et al., 2020
		Urine (pediatric inpatients)	*bla*_CTX-M-G1_ (61.1%), *bla*_CTX-M-G9_ (5.3%), *bla*_CTX-M-G2_ (1%), *bla*_CTX-M-G8_ (1%), *bla*_TEM_ (3.2%), *bla*_SHV_ (1%), *bla*_CTX-M-G1_ + *bla*_TEM_ (17.9%), *bla*_CTX-M-G9_ + *bla*_TEM_ (2.1%), *bla*_CTX-M-G8_ + *bla*_TEM_ (1%), *bla*_CTX-M-G1_ + *bla*_SHV_ (1%), *bla*_CTX-M-G1_ + *bla*_TEM_ + *bla*_SHV_ (5.3%)	Eltai et al., 2018b
Saudi Arabia	ESBL EC	Urine from UTI patients (in + outpatients); resistant to CTX/CLA and CAZ/CLA	*bla*_CTX-M_ + *bla*_TEM_ + *bla*_SHV_ (50%), *bla*_CTX-M_ + *bla*_SHV_ (50%)	Badger-Emeka et al., 2022
		Urine samples from UTI inpatients	*bla*_CTX-M-15_ (93.9%), *bla*_TEM_ (27.3%), *bla*_OXA_ (36.4%)	Alqasim et al., 2018
		Mixed	*bla*_TEM_ (44.4%, *bla*_TEM-1_ dominant), *bla*_CTX-M_ (33.3%, *bla*_CTX-M-15_ dominant), *bla*_CTX-M_ + *bla*_TEM_ (22.2%)	Ibrahim et al., 2021
		Mixed	**Beta-lactams:** *bla*_TEM-1B_ (63.2%), *bla*_SHV-12_ (5.3%), *bla*_OXA-1_ (31.6%), *bla*_CTX-M-3_ (5.3%), *bla*_CTX-M-8_ (5.3%), *bla*_CTX-M-15_ (78.9%), *bla*_CTX-M-27_ (5.3%), *bla*_CMY-42_ (5.3%), *bla*_DHA-1_ (5.3%); **Aminoglycosides**: *aph(3′)*-*la* (10.5%), *aph(3′′)*-*lb* (63.2%), *aph(6)*−*Id* (63.2%), *aac(3)*-*IIa* (26.3%), *aac(3)*-*VIa* (10.5%), *aac(6′)*-*Ib*-*cr* (31.6%), *aadA1* (26.3%), *aadA2* (15.8%), *aadA5* (10.5%), *aadA24* (5.3%) **Fluoroquinolone:** *qnrB4* (5.3%), *qnrS1* (21.1%), *qepA4*, (5.3%) **Macrolide/lincosamide/streptogramin**: *erm(B)* (21.1%), *mdf(A)* (100%), *mph(A)* (31.6%) **Phenicols**: *catA1* (5.3%), *catB3* (21.1%), *floR* (15.8%), *cmlA1* (5.3%) **Sulfonamide:** *sul1* (47.4%), *sul2* (63.2%), *sul3* (5.3%) **Tetracycline**: *tet(A)* (68.4%), *tet(B)* (10.5%) **Trimethoprim**: *dfrA1* (36.8%), *dfrA5* (5.3%), *dfrA12* (15.8%), *dfrA14* (26.3%), *dfrA17* (15.8%) **Fosfomycin:** *fosA3* (5.3%) **Fluoroquinolone resistance mutations:** *parC (S80I)* (63.2%), *parC (E84V)* (15.8%), *gyrA (S83L)* (73.7%), *gyrA (D87N)* (63.2%), *gyrA (DS87Y)* (5.3%), *parE (S458A)* (31.6%), *parE (I529L)* (10.5%), *parE (L416F)* (5.3%)	Yasir et al., 2020
		Mixed (neonatal ICU) (ST-131)	*bla*_CTX-M-15_ (100%), *bla*_SHV-2_ (47.4%), *bla*_SHV-12_ (15.8%), *bla*_TEM-1_ (68.4%)	Almogbel et al., 2021
		Blood	*bla*_CTX-M-15_ (66.7%), *bla*_CTX-M-14_ (13.3%), *bla*_TEM-1_ (53.3%), *bla*_OXA-1_ (33.3%)	Alangari et al., 2022
UAE	ESBL EC	Mixed	*bla*_CTX-M_ (100%) [*bla*_CTX-M-28_ (82.1%), *bla*_CTX-M-167_ (5.1%), *bla*_CTX-M-38, 163, and 198_ (2.6% each)], *bla_TEM_* (100%) [*bla*_TEM-171_ (20.5%), *bla*_TEM-206_ (5.1%), *bla*_TEM-120 and 163_ (2.6% each)], *bla*_SHV_ (38.5%) [*bla*_SHV-148 and 187_ (2.6% each)] and *bla*_VIM_ (5.1%)	Alfaresi et al., 2018
Kuwait	ESBL KP	Mixed (pediatric)	*bla*_CTX-M-15_ (84.2%), *bla*_CTX-M-14_ (2.6%), *bla*_CTX-M-27_ (1.3%), *bla*_CTX-M-209_ (1.3%), *bla*_CTX-M-9-type_ (1.3%), *bla*_SHV-2A_ (2.6%), *bla*_SHV-38_ (1.3%)	Al-Sweih et al., 2021
Qatar	ESBL KP	Urine (pediatric inpatients)	*bla*_CTX-M-G1_ + *bla*_TEM_ (15.4%), *bla*_TEM_ + *bla*_SHV_ (7.7%), *bla*_CTX-M-G1_ + *bla*_SHV_ (23.1%), *bla*_CTX-M-G1_ + *bla*_TEM_ + *bla*_SHV_ (46.2%), *bla*_CTX-M-G9_ + *bla*_TEM_ + *bla*_SHV_ (7.7%)	Eltai et al., 2018b
		Mixed (general pediatrics)	**ESBL genes:** *bla*_CTX-M-15_ (100%), *bla*_SHV-106_ (19.4%), *bla*_SHV-27_ (12.9%) **Other β-lactamase genes**: *bla*_TEM-1B_ (54.8%), *bla*_OXA-1_ (54.8%), *bla_NDM_*_1_*_/_*_5_ (12.9%), *bla*_DHA_ (6.5%), *bla*_OXA-48_ (3.2%) **Plasmid-mediated quinolone resistance genes**: *aac(6′)-Ib-cr* (45.1%), *qnr A/B/E/S* (67.8%) **Aminoglycoside-modifying enzyme genes**: *aac(3)-II [aac(3)-IIa, aac(3)-IId, aac(3)-IVa*, and *aac(3)-VIa*] *(35.5%), aadA1* (12.9%), *aadA2* (19.4%), *aadA5* (6.5%), *aph(3′)-Ia* (12.9%), *aph(3*^”^*)-Ib* (67.8%), *aph(6)-Id* (67.8%) **Cotrimoxazole resistance genes**: *dfrA/B* (80.7%), *sul1–3* (77.4%)	Perez-Lopez et al., 2020
		Mixed (neonatal ICU)	**ESBL genes:** *bla*_CTX-M-15_ (94.7%), *bla*_SHV-106_ (10.5%), *bla*_SHV-27_ (10.5%) **Other β-lactamase genes**: *bla*_TEM-1B_ (63.2%), *bla*_OXA-1_ (42.1) **Plasmid-mediated quinolone resistance genes**: *aac(6′)-Ib-cr* (36.7%), *qnr A/B/E/S* (57.9%) **Aminoglycoside-modifying enzyme genes**: *aac(3)-II [aac(3)-IIa, aac(3)-IId, aac(3)-IVa*, and *aac(3)-VIa*] (42.1%), *aadA1* (10.5%), *aadA2* (10.5%), *aph(3′)-Ia* (10.5%), *aph(3*^”^*)-Ib* (73.7%), *aph(6)-Id* (73.7%) **Cotrimoxazole resistance genes**: *dfrA/B* (63.2%), *sul1-3* (78.9%)	Perez-Lopez et al., 2020
		Mixed (pediatric ICU)	**ESBL genes:** *bla*_CTX-M-15_ (91.3%), *bla*_CTX-M-14_ (4.3%), *bla*_SHV-106_ (4.3%), *bla*_SHV-27_ (8.6%), *bla*_CTX-M-1_ (4.3%) **Other β-lactamase genes**: *bla*_TEM-1B_ (34.8%), *bla*_OXA-1_ (17.4%), *bla_NDM_*_1_*_/_*_5_ (4.3%), *bla*_DHA_ (4%), *bla*_TEM-33_*_/_*_35_ (4.3%) **Plasmid-mediated quinolone resistance genes**: *aac(6′)-Ib-cr* (17.4%), *qnr A/B/E/S* (73.9%) **Aminoglycoside-modifying enzyme genes**: *aac(3)-II [aac(3)-IIa, aac(3)-IId, aac(3)-IVa*, and *aac(3)-VIa*] (4.3%), *aadA1* (4.3%), *aadA2* (21.7%), *aadA5* (4.3%), *aph(3′)-Ia* (17.4%), *aph(3*^”^*)-Ib* (65.2%), *aph(6)-Id* (65.2%) **Cotrimoxazole resistance genes**: *dfrA/B* (60.9%), *sul1-3* (60.9%)	Perez-Lopez et al., 2020
		Mixed from ICU	*bla*_CTX-M [mainly CTX-M-1]_ (75%), *bla*_TEM_ (53.6%), *bla*_SHV_ (87.5%), *bla*_CTX-M_ + *bla*_TEM_ + *bla*_SHV_ (46.4%), *bla*_CTX-M_ + *bla*_TEM_ (7.1%), *bla*_CTX-M_ + *bla*_SHV_ (17.8%)	Sid Ahmed et al., 2016
Saudi Arabia	ESBL KP	Mixed	*bla*_TEM_ (60.7%, *bla*_TEM-1_ dominant), *bla*_CTX-M_ (7.1%, *bla*_CTX-M-15_ dominant), *bla*_OXA-1_ (3.6%), *bla*_CTX-M_ + *bla*_TEM_ (25%), *bla*_TEM_ + *bla*_SHV_ (3.6%). *bla*_CTX-M-3,_ *bla*_OXA-1_ and *bla*_OXA-232_ also noted.	Ibrahim et al., 2021; Moglad et al., 2022
		Mixed from mostly healthcare-associated admitted patients	*bla*_TEM_ (4.3%, *bla*_TEM-1_ dominant), *bla*_CTX-M_ (8.7%, *bla*_CTX-M-15_ dominant), *bla*_SHV_ (34%, SHV-9 dominant), *bla*_CTX-M_ + *bla*_TEM_ (13%), *bla*_CTX-M_ + *bla*_SHV_ (13%), *bla*_TEM_ + *bla*_SHV_ (8.7%), *bla*_KPC_ (13%, KPC-2 and 3 dominant), *bla*_IMP_ (8.7%, IMP4 dominant)	Azim et al., 2019
		Mixed (neonatal ICU) (ST-14)	*bla*_CTX-M-15_ (92.8%), *bla_SHV-12_* (92.8%), *bla*_TEM-1_ (88%). High co-expression of *bla*_CTX-M-15_ and *bla_SHV-12_* (90.5%) resulting in high-level resistance to oxyimino-cephalosporins	Almogbel et al., 2021
	Colistin-resistant MDR KP	Mixed samples from inpatients	**Beta-lactams:** *bla*_AmpH_ (100%), *bla*_TEM-150_ (70%), *bla*_OXA-1_ (100%), *bla*_OXA-181_ (70%), *bla*_SHV-106_ (80%), *bla*_SHV-110_ (20%), *bla*_CTX-M-15_ (90%), *bla*_CTX-M-9_ (10%), *bla*_OXA-48_ (30%); **Aminoglycosides**: *pbp* (100%), *aadA2* (70%), *ant(3′)-lh* (100%), *aac(3)-IIa* (20%), *aph(3′′)-Vlb* (20%), *armA* (40%), *StrAB* (20%) **Streptothricin:** *Sat-2A* (80%) **Fluoroquinolone:** *OqxA* (100%), *OqxBgb* (100%), *qnrB1* (20%) **Macrolide:** *mph(D)* (90%); *mph(E)* (60%); *msr(E)* (70%) **Phenicols**: *catA1* (30%), *catB4* (100%) **Fosfomycin:** *fosA2* (100%) **Sulfonamide**: *sul1* (90%) **Tetracycline**: *tet(34)* (100%), *tet(A)* (20%), *tet(D)* (50%) **Trimethoprim**: *dfrA12* (70%), *dfrA14* (10%) **Colistin:** *mcr-1* (20%), *mgrB* [Q22P (20%), ΔMgrB(20%)], *pmrA* [G53V (10%)], *pmrB* [T128P; T157P (10%) each], *phoQ* [L52V (10%), G385S (10%), D495 (10%), L52V (10%) each]	Okdah et al., 2022
	Carbapenem-resistant KP	Mixed samples	*Bla*_CTX-M-3_ (25%), *Bla*_CTX-M-57_ (25%), *Bla*_CTX-M-82_ (25%), *Bla*_CTX-M-15_ (25%), *bla*_TEM-1_ (100%), *bla*_OXA-1_ (100%), *rmtC* (75%)*, aac(6)-Ib* (100%), *qnrB* (75%)	Al-Agamy et al., 2019
UAE	ESBL KP	Mixed	*bla*_CTX-M_ (100%) [*bla*_CTX-M-28_ (80%)], *bla*_TEM_ (100%), *bla*_SHV_ (80%)	Alfaresi et al., 2018

Mixed source indicates all clinical specimens, including lower respiratory tract infections, blood, urine, wound infections, etc.

AMK, amikacin; AMP, ampicillin; AMX/CLV, amoxicillin/clavulanate; AZT, aztreonam; CAZ/CLA, ceftazidime/clavulanic acid; CFP, cefepime; CFT, cefotaxime; CFX, cefixime; CFZ, ceftazidime; CFZO, cefazolin; CLT, cephalothin; COL, colistin CTX, ceftriaxone; CTX/CLA, cefotaxime/clavulanic acid; CX, cefoxitin; CXM, cefuroxime; EAEC, enteroaggregative Escherichia coli; EPEC, enteropathogenic Escherichia coli; ERP, ertapenem; FQ, fluoroquinolone (ciprofloxacin/norfloxacin/levofloxacin); GEN, gentamicin; IMP, imipenem; KP, Klebsiella pneumoniae; MNM, meropenem; NF, nitrofurantoin; PIP/TAZ, piperacillin/tazobactam; TG, tigecycline; TMPS, trimethoprim−sulfamethoxazole; UAE, United Arab Emirates. Ranked by country and organism. % represents prevalence rates.

Previously, a meta-analysis of studies from the GCC region on *E. coli* strains collected between 2013 and 2019 from the region identified that the main resistance genes in the region included CTX−M (53.8%), followed by TEM (40.6%), NDM−1 (28.4%), OXA (24.3%), VIM (8.5%), and SHV (7.8%) (Bindayna et al., 2022). As evidenced in **Table 2**, this is largely still the case for ESBL *E. coli* and *K. pneumoniae*, where CTX−M dominates (55.5%–100% and 32.1%–100%, respectively), followed by TEM (27.3%–100% and 26%–100%, respectively), and SHV (5.3%–100% and 3.6%–100%, respectively) (**Table 2**).

### 3.4 Prevention measures

One of the main aims of introducing antimicrobial stewardship programmes (ASPs) in healthcare is to combat the growing rates of AMR. Over the last decade, most GCC countries introduced ASPs in healthcare settings, with various implementation challenges (McGowan and Gerding, 1996; Hashad et al., 2020). In some countries in the region, the implementation of ASPs is showing success, as evidenced by the incidence of MDR *Enterobacteriaceae*, which have declined in response to strict implementation of ASPs in Bahrain. The adoption of strict policies in 2014 in Bahrain led to a decrease in the incidence of MDR *Enterobacteriaceae* from a high of 45.4 cases per 10,000 patient admissions to 23.2 cases per 10,000 patient admissions (Saeed et al., 2019). In addition, to combat the increasing AMR threat, many countries in the world, including GCC countries, have adopted the Global Action Plan on AMR (GAPAMR), the implementation of which is monitored by the Tracking AMR Country Self-Assessment Survey (TrACSS 2022 country reports, 2022). Saudi Arabia and Oman have demonstrated nationwide implementation of most indicators of national progress and capacity on tackling AMR, at levels exceeding the global average for many of these indicators. Regionally, Qatar has achieved nationwide implementation of three of the five indicators in the human health sector. Kuwait, the United Arab Emirates, and Bahrain have demonstrated success in implementing two of the five indicators. Although substantial progress has been made in tackling AMR in the GCC region in the last few years, this still remains an area of concern and needs continuous attention and efforts.

### 3.5 Limitations

One of the fundamental limitations of this review is that the prevalence of MDR, microbiological characteristics, the distribution of isolates, and ESBL activity are very difficult to compare between studies because of the paucity of reports from certain countries and because of the heterogeneity in studies, with specific differences in sample sources (i.e., community, hospital outpatient/inpatients, and critical care), sample types (i.e., urine, respiratory, wound and bloodstream infections), collection periods, regional and local guidelines for classifying intermediate resistance as resistant/susceptible, and type of analytical methods used (Bandy and Tantry, 2021). In addition, there exists inherent limitations in individual studies, including small sample sizes and studied populations. These differences make comparisons between studies susceptible to conclusive biases. Nevertheless, the review compiled the majority of its data from recent and available published literature in the studied subject from all regional countries, which will hopefully pave the way for future research.

## 4 Summary and conclusions

The GCC countries have a substantial expatriate population, ranging from approx 40-90%. The region is an important destination for travel, tourism, and global trade, and frequently hosts international or religious events, such as Umrah and Hajj. These factors, along with an embedded culture of inappropriate, excessive antimicrobial prescribing, and poor antibiotic regulations, spearhead the propagation of AMR (Sonnevend et al., 2015; Saeed et al., 2019).

The prevalence rates of ESBL-*Enterobacterales* in the GCC region predominated by *E. coli* and *K. pneumoniae* are similar to many global regions with intermediate regional prevalence; however, higher prevalence rates were observed in patients with UTIs and in samples from critical care. The prevalence of ESBL in the community, including carrier state, is alarming, and, therefore, there is a need for critical surveillance, exploration of underlying precipitating factors, and development of public health preventive mechanisms to halt its spread.

Owing to the increased morbidity, mortality, and economic costs associated with MDR infections, predictors for the acquisition of MDR infections would be invaluable tools to assist its management (Baiou et al., 2021; El-Sokkary et al., 2021). Another area of focus should be the development of novel therapeutics against these organisms, an area that has been significantly neglected in the past decade. A distinctive fact is that *Enterobacterales* isolates that are ESBL producing and carbapenem resistant are being increasingly reported in the region, which warrants concomitant control and prevention measures, and the introduction of novel therapeutic agents.

Promisingly, however, there is a growing research focus in this field in almost all GCC countries, which should strengthen the monitoring process in these countries. Furthermore, strict antimicrobial stewardship is also being implemented in almost all GCC countries, which will hopefully control and monitor the appropriate prescribing of antimicrobials on a long-term basis.

## Author contributions

HH, HA-H, LA, MA-O, MAS, HH, HM, BS, AS, and SS analyzed the data and wrote/reviewed the manuscript. All authors contributed to the article and approved the submitted version.
