# Supplementary material for: Prevalence and genetic characterization of clinically relevant extended-spectrum β-lactamase-producing Enterobacterales in the Gulf Cooperation Council countries
Source: Front Antibiot. 2023 Jun 26;2:1177954. doi: 10.3389/frabi.2023.1177954 (PMC11732020; doi:10.3389/frabi.2023.1177954)
Supplement: Supplementary file 1 [file DataSheet_1.docx]

Supplementary Table 1: **ESBL *E. coli* prevalence rate in GCC and antimicrobial resistance profile** Ranked by country and source. % represents resistance rates.

| **Country** | **Number of ESBL *EC*** | **Source** | **AMP** % | **AMX/CLV** % | **PIP/TAZ %** | **CLT** % | **CX** % | **CFT** % | **CFX**% | **CXM** % | **CFZO** % | **CTX** % | **CFZ** % | **CFP** % | **ERP** % | **MNM** % | **IMP** % | **NF** % | **GEN** % | **AMK** % | **TMPS** % | **FQ** % | **FOS** % | **TG** % | **AZT** % | **COL** % | **Reference(s)** |
| --- | --- | --- | --- | --- | --- | --- | --- | --- | --- | --- | --- | --- | --- | --- | --- | --- | --- | --- | --- | --- | --- | --- | --- | --- | --- | --- | --- |
| Bahrain | 32.9% among *EC* cases | Mixed clinical specimens | - | 97.3 | 11 | - | - | - | 97.3 | 98.6 | 94.5 | 97.9 | 96.6 | 84.2 | 2.1 | 1.4 | 1.4 | - | 27.4 | 1.4 | 52.1 | 50.7-52.1 | - | - | - | - | (Kasim O. Ardati, 2019) |
| Kuwait | 71.3% (non-CRE) among *EC* | Mixed | - | - | - | - | - | - | - | - | - | - | - | - | - | - | - | - | - | - | - | - | - | - | - | - | (Karlowsky et al., 2022) |
|  | 40% among *EC* | Mixed (Pediatric) | - | 17.7 | 3.2 | - | - | - | - | - | - | - | 62.9 | 75.8 | - | 1.6 | 1.6 | - | - | 0 | - | 46.8 | - | 0 | 79 | 0 | (Al-Sweih et al., 2021) |
| Oman | 37% among *EC* | Mixed | - | - | 5 | - | - | - | - | - | - | - | - | - | - | 20 | 1 | 5 | 20 | 3 | - | - | - | - | - | - | (Al Rahmany et al., 2019) |
| Qatar | 34.7% among ESBL *EB* | Mixed (ICU) | - | - | - | - | - | - | - | - | - | - | - | - | - | 0 | - | - | - | - | - | - | - | - | - | - | (Sid Ahmed et al., 2016) |
|  | 48.9% (non-CRE) among *EC* | Mixed | - | - | - | - | - | - | - | - | - | - | - | - | - | - | - | - | - | - | - | - | - | - | - | - | (Karlowsky et al., 2022) |
|  | - | Mixed (pediatric population) | - | 36.5 | 15.4 | - | - | - | - | - | - | 100 | 63.5 | 92.3 | 1.9 | 0 | - | 0 | 25 | 1.9 | 61.5 | 44.2-48.1 | - | - | 83.7 | - | (Perez-Lopez et al., 2020) |
|  | - | Rectal swab screening  for carbapenemase-producers (pediatric patients requiring surgery/hospitalization) | - | 48.5 | 11.9 | - | - | - | - | - | - | 99 | 58.9 | 62.4 | 5.9 | 3.9 | - | 9.9 | 16.3 | 0.5 | 66.8 | 39.6-41.8 | - | - | 84.4 | 1 | (Perez-Lopez et al., 2020) |
| Saudi Arabia | 37.1% among *EC* causing healthcare associated infections | Mixed (2015-2019) | - | - | - | - | - | - | - | - | - | - | - | - | - | - | - | - | - | - | - | - | - | - | - | - | (Mutair et al., 2021) |
|  | 20% among *EC* | Mixed (2013-2018) | - | - | 10.1 | - | 24.5 | - | - | - | - | - | 68.5 | 27.8 |  | 0.2 | 7.8 | 16.3 | 28.8 | 1.3 | 65.6 | 73.4 | - | - | - | - | (Al-Tawfiq et al., 2020) |
|  | 37.5% of all *EC* infections (ICU) | Mixed samples from ICU patients | - | - | - | - |  | - | - | - | - | - | - | - | - | - | - | - | - | - | - | - | - | - | - | - | (Kabrah et al., 2021) |
|  | 44.4% among ESBL gram negative bacteria/*EB* | Mixed samples from ICU inpatients with central venous catheter | 100 | 18.7 | - | - | - | 100 | - | 100 | - | - | 100 | 100 | - | - | 0 | - | 25 | - | 56.2 | 56.2 | - | - | - | - | (Kabrah, 2022) |
|  | 34-66.4% among ESBL *EB*, 36.6% among *EC* | Mixed | - | - | - | - | - | - | - | - | - | - | - | - | 6.2 | 1.4 | 2.7 | - | 21.2 | 13.1 | 68.7 | 69-70.8 | - | 4.1 | 97.3 | - | (Ibrahim et al., 2021, Bandy and Tantry, 2021, Aldrazi et al., 2020) |
|  | *31.1%* among *EC* | Mixed (inpatients) | - | - | - | - | - | - | - | - | - | - | - | - | - | - | - | - | - | - | - | - | - | - | - | - | (Ibrahim et al., 2019) |
|  | 60% among CRE *EC,* (carbapenem resistant) | Mixed CRE isolates from inpatients | - | - | 100 | - | 33.3 | 100 | - | - | - | - | 100 | 100 | 33.3 | 0 | 33.3 | - | 50 | 0 | - | 100 | 0 | 0 | 100 | 0 | (Al-Agamy et al., 2018) |
|  | 62.7% among GNB | Mixed | 100 | 9-42.1 | 0-3 | 100 | 10.5 | 100 | - | 100 | 100 | 100 | 100 | 100 | - | 0 | 0 | 1-31.6 | 26.3-32 | 1-15.8 | 78.9 | 63.2-77 |  | 0 | 100 | - | (Yasir et al., 2020, Al-Garni et al., 2018) |
|  | *79.2%* among *EC (ST131)* | Mixed (neonatal ICU) | 94.7 | - | - | - | - | - | - | - | - | - | 94.7 | - | - | - | 0 | - | 0 | 0 | - | 0 | - | - | - | - | (Almogbel et al., 2021) |
|  | 44.4% of *EC* resistant to third generation cephalosporins | Mixed | - | - | - | - | - | - | - | - | - | - | - | - | - | - | - | - | - | - | - | - | - | - | - | - | (Azab et al., 2021) |
| **Country** | **Number of ESBL *EC*** | **Source** | **AMP** % | **AMX/CLV** % | **PIP/TAZ** % | **CLT** % | **CX** % | **CFT** % | **CFX** % | **CXM** % | **CFZO** % | **CTX** % | **CFZ** % | **CFP** % | **ERP** % | **MNM** % | **IMP** % | **NF** % | **GEN** % | **AMK** % | **TMPS** % | **FQ** % | **FOS** % | **TG** % | **AZT** % | **COL** % | **Reference(s)** |
| UAE | 86.6% among ESBL *EB*, 38.6% ESBL non-CRE *EC* among total *EC* | Mixed | 97.4 | 5.1 | 2.6 | - | - | 97.4 | - | - | - | - | 97.4 | 97.4 | 0 | 0 | 0 | 2.6 | 10.3 | 0 | 59 | 46.2-59 | 0 | - | - | - | (Alfaresi et al., 2018, Karlowsky et al., 2022) |
| Kuwait | 18.8% among *EC* isolates from food handlers (of these, 71.2% were healthcare vs. 28.8% were community food handlers, p=0.001) | Fecal samples from food handlers | - | - | - | - | - | - | - | - | - | - | - | - | - | - | - | - | - | - | - | - | - | - | - | - | (Moghnia et al., 2021) |
| Oman | 75% among MDR *EC* | Endotracheal specimens, ventilated patients | - | - | - | - | - | - | - | - | - | - | - | - | - | - | - | - | - | - | - | - | - | - | - | - | (Sannathimmappa et al., 2021) |
| Qatar | 22.4% among EPEC and EAEC | Fecal  (Pediatric patients with acute gastroenteritis) | - | - | - | - | - | - | - | - | - | - | - | - | - | - | - | - | - | - | - | - | - | - | - | - | (Eltai et al., 2020) |
|  | 9% among commensal *EC* | Fecal (healthy food handlers) | - | - | - | - | - | - | - | - | - | - | - | - | - | - | - | - | - | - | - | - | - | - | - | - | (Eltai et al., 2018c) |
| Saudi Arabia | 52.2% among *EC* | Blood stream infection-hospitalized patients | - | - | - | - | - | - | - | - | - | - | - | - | - | 0 | - | - | - | - | - | - | - | 0 | - | 0 | (Bandy and Almaeen, 2020) |
|  | 3.9% of all respiratory infections and 100% of respiratory *EC* infections among ICU patients | Respiratory samples from ICU patients | - | - | - | - | - | - | - | - | - | - | - | - | - | - | - | - | - | - | - | - | - | - | - | - | (Kabrah et al., 2021) |
|  | 24.1% of *EC* causing emphysematous pyelonephritis | Patients with emphysematous pyelonephritis | - | - | - | - | - | - | - | - | - | - | - | - | - | - | - | - | - | - | - | - | - | - | - | - | (Robles-Torres et al., 2022) |
| Bahrain | 23.49-50% among *EC* cases | Urine from pediatric inpatients with confirmed UTI | 100 | 86.2-100 | 9.7 | 100 | - | 100 | - | 96-100 | 100 | 97-100 | 97 | - | 0* | | | 0 | 13.7 | 3.03 | 55.9 | 25.8 | - | 0 | - | - | (Shaaban et al., 2021, Mohammed et al., 2022) |
|  | 38.1% overall (37.3%i in 2018 and 39% among *EC* cases in 2019) | Urine | - | 100 | - | - | - | - | - | - | - | - | - | - | - | - | - | 5.7-10.7 | - | - | 49.8-58.1 | 50.5-51.6 | 1.2-3.7 |  | - | - | (Saeed et al., 2021) |
| **Country** | **Number of ESBL *EC*** | **Source** | **AMP** % | **AMX/CLV** % | **PIP/TAZ** % | **CLT** % | **CX** % | **CFT** % | **CFX** % | **CXM** % | **CFZO** % | **CTX** % | **CFZ** % | **CFP** % | **ERP** % | **MNM** % | **IMP** % | **NF** % | **GEN** % | **AMK** % | **TMPS** % | **FQ** % | **FOS** % | **TG** % | **AZT** % | **COL** % | **Reference(s)** |
| Oman | 26% among uropathogenic *EC* | Urine from patients with complicated UTI | - | - | - | - | - | - | - | - | - | - | - | - | - | - | - | - | - | - | - | - | - | - | - | - | (Al Mamari et al., 2022) |
| Kuwait | 26.4% among uropathogenic *EC* | Urine from patients with UTI (pediatric) | - | 63 | 9 | - | - | - | - | - | - | - | - | 74 | - | 0 | - | 5 | 20 | 2 | 65 | 27 | - | - | - | - | (Al Benwan and Jamal, 2022) |
| Qatar | 79% among ESBL EB | Urine from UTI patients | - | - | - | - | - | - | - | - | - | - | - | - | - | - | - | - | - | - | - | - | - | - | - | - | (Naushad et al., 2022) |
| Saudi Arabia | 11.9 % among UTI infections among pediatric patients | Urine samples from UTI pediatric inpatients | 100 | 33.3 | - | 100 | 100 | - | - | 100 | - | 100 | 100 | 100 | - | 0 | 0 | 0 | 63.6 | 7.7 | 33.3 | 0-100 | - | - | 100 | - | (Alzahrani et al., 2021) |
|  | 4-33.5% among uropathogenic *EC,* 10.32-11.8% among all uropathogens, 66.3% in females and 33.7% in males | Urine samples from UTI patients (inpatients + outpatients) | 47.8-100 | 55-89.4 | 3-27.1 | 99.8 | 0-16 | 99.6 | - | 99.4-100 | 100 | 99.6 | 98.4-100 | 0-100 | 0 | 0-0.4 | 0-0.1 | 5-52.2 | 27.5-52.2 | 0-1 | 34.8-65 | 0-69 | - | 34.8-77.1 | 99.2 | 58.4 | (Bazaid et al., 2021, Alzahrani et al., 2020, Badger-Emeka et al., 2022, Balkhi et al., 2018, Abalkhail et al., 2022) |
|  | Elderly: 8.33%, Adults: 4.82 and pediatric: 3.23% among uropathogens | Urine samples from UTI patients (emergency department) | - | - | - | - | - | - | - | - | - | - | - | - | - | - | - | - | - | - | - | - | - | - | - | - | (Alanazi et al., 2018) |
|  | 30-33% among uropathogenic *EC* | Urine samples from UTI patients (inpatients) | 100 | 88 | - | - | 18 | - | - | - | - | - | 85 | - | - | - | 0 | 21 | 27 | - | 82 | 76 | - | - | - | - | (Alqasim et al., 2018, Abdallah et al., 2020) |
|  | 5.9% of all urinary infections and 25% of uropathogenic *EC* in ICU patients | Urine samples from ICU patients | - | - | - | - | - | - | - | - | - | - | - | - | - | - | - | - | - | - | - | - | - | - | - | - | (Kabrah et al., 2021) |
|  | 6.5% of all uropathogens, 11.1% of *EC* causing UTI | Urine samples from female outpatients | - | - | - | - | - | - | - | - | - | - | - | - | 0 | - | - | 0 | - | 0 | - | - | - | - | - | - | (Alasmary, 2021) |
| UAE | 23% among *EC* | Urine samples from UTI patients from community | 100 | - | - | - | - | - | - | - | - | - | - | - | - | 0 | - | - | - | - | - | - | - | - | - | - | (Ranjan Dash et al., 2018) |

Mixed source indicates all clinical specimens including lower respiratory tract infections, blood, urine, wound infections etc. AMP: Ampicillin, AMX/CLV: Amoxicillin/clavulanate, CLT: Cephalothin, CX: Cefoxitin, CFT: Cefotaxime, CFZ: Ceftazidime, CFP: Cefepime, CFZO: Cefazolin, CFX: Cefixime; CXM: Cefuroxime, CTX: Ceftriaxone, ERP: Ertapenem, MNM: Meropenem, IMP: Imipenem, GEN: Gentamicin, AMK: Amikacin, TMPS: Trimethoprim‑sulfamethoxazole, FQ: Fluoroquinolone (Ciprofloxacin/Norfloxacin/Levofloxacin/Moxifloxacin), FOS: Fosfomycin, NF: Nitrofurantoin, PIP/TAZ: Piperacillin/tazobactam, TG: Tigecycline, AZT: Aztreonam, COL: Colistin. - Not tested/not available. XDR: Extensively drug resistant, PDR: Pan drug resistant. *EC: E. coli,* EPEC: Enteropathogenic *EC,* EAEC= Enteroaggregative *EC.* *drug not specified. - not reported.

Supplementary Table 2: **ESBL *K. pneumoniae* prevalence rate in GCC and antimicrobial resistance profile**. Ranked by country and source. % represents resistance

| **Country** | **Number of resistant isolates** | **Source** | **AMP** % | **AMX/CLV** % | **PIP/TAZ** % | **CX** % | **CFT** % | **CFX** % | **CXM** % | **CFZO** % | **CTX** % | **CFZ**% | **CFP** % | **ERP** % | **MNM** % | **IMP** % | **NF** % | **GEN** % | **AMK** % | **TMPS** % | **FQ** % | **AZT** % | **COL**% | **FOS** % | **TG** % | **Reference(s)** |
| --- | --- | --- | --- | --- | --- | --- | --- | --- | --- | --- | --- | --- | --- | --- | --- | --- | --- | --- | --- | --- | --- | --- | --- | --- | --- | --- |
| Bahrain | 18.3% among *KP* | Mixed clinical specimens | - | 89.7 | 23.1 | - | - | 87.2 | 89.7 | 82.1 | 89.7 | 87.2 | 84.6 | 10.3 | 7.7 | 7.7 | - | 46.2 | 12.8 | 38.5 | 41-48.7 | - | - | - | - | (Kasim O. Ardati, 2019) |
| Kuwait | 52.4% (non CRE) among *KP* | Mixed | - | - | - | - | - | - | - | - | - | - | - | - | - | - | - | - | - | - | - | - | - | - | - | (Karlowsky et al., 2022) |
|  | 31.5% among hospital acquired (ICU) *KP* infections | Mixed | - | - | - | - | - | - | - | - | - | - | - | - | - | - | - | - | - | - | - | - | - | - | - | (Alfouzan et al., 2021) |
|  | 53.1% among *KP* | Mixed (Pediatric) | - | 36.8 | 26.3 | - | - | - | - | - | - | 85.5 | 65.8 | - | 1.3 | 0 | - | - | 3.9 | - | 15.8 | 88.2 | 2.7 | - | 0 | (Al-Sweih et al., 2021) |
| Oman | 23% among *KP* | Mixed | - | - | 20 | - | - | - | - | - | - | - | - | - | - | 1 | 28 | 45 | 7 | - | 25 | - | - | - | 0 | (Al Rahmany et al., 2019) |
| Qatar | 51.4% among ESBL *EB* | Mixed (ICU) | - | - | - | - | - | - | - | - | - | - | - | - | 0 | - | - | - | - | - | - | - | - | - | - | (Sid Ahmed et al., 2016) |
|  | 28.2% (non CRE) among *KP* | Mixed | - | - | - | - | - | - | - | - | - | - | - | - | - | - | - | - | - | - | - | - | - | - | - | (Karlowsky et al., 2022) |
|  | - | Mixed (pediatric population) | - | 58.8 | 35.3 | - | - | - | - | - | 100 | 100 | 100 | 5.9 | 5.9 | - | 41.2 | 35.3 | 0 | 88.2 | 11.8-47.1 | 100 | - | - | - | (Perez-Lopez et al., 2020) |
|  | - | Rectal swab screening  for carbapenemase-producing organism (pediatric patients requiring surgery/hospitalization) | - | 76.8 | 28.6 | - | - | - | - | - | 98.2 | 83.9 | 66.1 | 10.7 | 8.9 | - | 64.3 | 28.6 | 5.4 | 73.2 | 20-39.3 | 89.7 | - | - | - | (Perez-Lopez et al., 2020) |
| Saudi Arabia | ESBL *Klebsiella spp*: 27.8% among *Klebsiella spp* causing HAI | Mixed (2015-2019) | - | - | - | - | - | - | - | - | - | - | - | - | - | - | - | - | - | - | - | - | - | - | - | (Mutair et al., 2021) |
|  | 26% among *KP* | Mixed (2013-2018) | - | 59.5 | 32.5 | 16.4 | - | - | - | - | - | 77.9 | 23.4 | - | 2.2 | 2 | 81.6 | 37.5 | 2.4 | 77.7 | 56.4 | - | - | - | - | (Al-Tawfiq et al., 2020) |
|  | 21.3 % among *KP* | Mixed (2011-2021) | >90 | - | - | - | >90 | - | - | - | - | >90 | >90 | - | - | 14.63 ±18.2 | - | - | 21.3±17.4 | - | - | - | - | - | 19.67±23 | (Jalal et al., 2023) |
|  | 7% of all *KP* infections in ICU patients | Mixed (ICU patients) | - | - | - | - | - | - | - | - | - | - | - | - | - | - | - | - | - | - | - | - | - | - | - | (Kabrah et al., 2021) |
|  | 41.6% among ESBL gram negative bacteria | Mixed samples from ICU inpatients with central venous catheter | 93.3 | 20 | - | - | 93.3 | - | 93.3 | - | - | 93.3 | 93.3 | - | - | 6.6 | - | 46.6 | - | 93.3 | 53.3 | - | - | - | - | (Kabrah, 2022) |
|  | 56.4 % among *KP* | Mixed samples from ICU (suspected HAI) | - | - | - | - | - | - | - | - | - | - | - | - | - | - | - | - | - | - | - | - | - | - | - | (Saleem et al., 2023) |
|  | **Number of resistant isolates** | **Source** | **AMP** % | **AMX/CLV** % | **PIP/TAZ** % | **CX** % | **CFT** % | **CFX** % | **CXM** % | **CFZO** % | **CTX** % | **CFZ** % | **CFP**% | **ERP** % | **MNM** % | **IMP** % | **NF** % | **GEN** % | **AMK** % | **TMPS** % | **FQ** % | **AZT** % | **COL** % | **FOS** % | **TG** % | **Reference(s)** |
|  | 20.3-52.8% among ESBL *EB*, 13 % among *KP,* 98% among MDR *KP; 23.6*% among GNB | Mixed | 100 | 24 | 15 | - | 100 |  | 98 | 100 | - | 100 | 100 | 3.9 | 0-1 | 0 | 39 | 44-56.7 | 10-14.4 | 68 | 30.8-48 | 100 | - | - | 8-23.4 | (Ibrahim et al., 2021, Bandy and Tantry, 2021, Badger-Emeka et al., 2021, Aldrazi et al., 2020, Al-Garni et al., 2018) |
|  | - | Mixed from mostly HAI, inpatients | 100 | 56.5 | 34.8 | - | 100 | - | - | - | 100 | 100 | 100 | - | - | 8.7  - | - | 34.8 | 8.7 | - | 43.5 | - | - | - | - | (Azim et al., 2019) |
|  | 35.71-41.2 % among *KP* | Mixed (inpatients) | 100 | - | - | - | - | - | - | - | - | - | - | - | - | - | - | - | - | - | - | - | - | - | - | (Ibrahim et al., 2019, Lagha et al., 2021) |
|  | 57.5-77.8% of *KP* resistant to third and fourth generation cephalosporins | Mixed (inpatients) (2014-2018) | - | - | - | - | - | - | - | - | - | - | - | - | - | - | - | - | - | - | - | - | - | - | - | (Al-Zalabani et al., 2020) |
|  | 50% of *KP* resistant to third generation cephalosporins | Mixed | - | - | - | - | - | - | - | - | - | - | - | - | - | - | - | - | - | - | - | - | - | - | - | (Azab et al., 2021) |
|  | 90.5% among CRE *KP* (carbapenem resistant) | Mixed CRE isolates (inpatients) | - | - | 100 | 100 | 100 | - | - | - | - | 94.7 | 94.7 | 47.4 | 26.3 | 42.1 | - | 73.7 | 15.8 | - | 89.5 | 89.5 | 5.3 | 0 | 0 | (Al-Agamy et al., 2018) |
|  | 87.5 % among *KP* | Mixed (neonatal ICU) | 100 | - | - | 100 | - | - | - | - | - | 97.1 | - | - | - | 0 | - | 90 | 0 | - | 0 | - | - | - | - | (Almogbel et al., 2021) |
| UAE | 11.1% among ESBL *EB*, 20.8% ESBL non CRE *KP* among all *KP* | Mixed | 100 | 20 | 0 | - | 100 | - | - | - | - | 100 | 100 | 0 | 0 | 0 | 0 | 60 | 0 | 40 | 0-20 | - | - | 0 | - | (Alfaresi et al., 2018, Karlowsky et al., 2022) |
| Kuwait | 4% among *KP* isolates from food handlers, 100% of these were healthcare food handlers | Fecal samples from food handlers | - | - | - | - | - | - | - | - | - | - | - | - | - | - | - | - | - | - | - | - | - | - | - | (Moghnia et al., 2021) |
| Oman | 17.1% among MDR *KP* | Endotracheal specimens, ventilated patients | - | - | - | - | - | - | - | - | - | - | - | - | - | - | - | - | - | - | - | - | - | - | - | (Sannathimmappa et al., 2021) |
| Saudi Arabia | 3.6% of all blood infections and 10% of *KP* causing blood infections | Blood (ICU patients) | - | - | - | - | - | - | - | - | - | - | - | - | - | - | - | - | - | - | - | - | - | - | - | (Kabrah et al., 2021) |
|  | 19% among *KP* | Blood stream infections-hospitalized patients | 100 | - | - | - | - | - | - | - | - | - | - | - | - | - | - | - | - | - | - | - | - | - | - | (Bandy and Almaeen, 2020) |
|  | **Number of resistant isolates** | **Source** | **AMP** % | **AMX/CLV** % | **PIP/TAZ** % | **CX** % | **CFT** % | **CFX** % | **CXM** % | **CFZO** % | **CTX** % | **CFZ** % | **CFP** % | **ERP** % | **MNM** % | **IMP** % | **NF** % | **GEN** % | **AMK** % | **TMPS** % | **FQ** % | **AZT** % | **COL** % | **FOS** % | **TG** % | **Reference(s)** |
|  | 22.2% among septic UTI infections, 100% of *KP* causing blood infections | Blood samples from UTI patients in NICU | 50 | - | - | 50 | - | - | - | - | - | - | 100 | - | 0 | 0 | 0 | 50 | 50 | 0 | 0 | - | - | - | 0 | (Bazaid et al., 2022) |
|  | 3.9% of respiratory infections and 5.5% of *KP* causing respiratory infections | Respiratory samples (ICU patients) | - | - | - | - | - | - | - | - | - | - | - | - | - | - | - | - | - | - | - | - | - | - | - | (Kabrah et al., 2021) |
|  | ESBL *Klebsiella spp*: 25% among *Klebsiella spp* | Surgical site infections | - | - | - | - | - | - | - | - | - | - | - | - | - | - | - | - | - | - | - | - | - | - | - | (El-Saed et al., 2020) |
|  | ESBL *Klebsiella spp*: 34.3% among *Klebsiella spp* | Device associated healthcare infections | - | - | - | - | - | - | - | - | - | - | - | - | - | - | - | - | - | - | - | - | - | - | - | (Balkhy et al., 2020) |
|  | 30% of *KP* causing emphysematous pyelonephritis | Patients with emphysematous pyelonephritis | - | - | - | - | - | - | - | - | - | - | - | - | - | - | - | - | - | - | - | - | - | - | - | (Robles-Torres et al., 2022) |
| Bahrain | 39.5% among *KP* | Urine (infant inpatients with UTI) | 100 | 100 | - | - | 100 | - | 100 | - | 100 | - | - | 0* | | | - | - | - | - | - | - | - | - | 0 | (Mohammed et al., 2022) |
| Oman | 18% among uropathogenic *KP* | Urine from patients with complicated UTI | - | - | - | - | - | - | - | - | - | - | - | - | - | - | - | - | - | - | - | - | - | - | - | (Al Mamari et al., 2022) |
| Kuwait | 35.3% among uropathogenic *KP* | Urine from patients with UTI (pediatric) | - | 72 | 17 | - | - | - | - | - | - | - | 60 | - | 0 | - | 57 | 22 | 14 | 45 | 31 | - | - | - | - | (Al Benwan and Jamal, 2022) |
| Qatar | 18% among ESBL EB | Urine from UTI patients | - | - | - | - | - | - | - | - | - | - | - | - | - | - | - | - | - | - | - | - | - | - | - | (Naushad et al., 2022) |
| Saudi Arabia | 13% among UTI infections; 29.2% among *KP* (UTI) | Urine samples from UTI patients in NICU | 71 | - | - | 14 | - | - | - | - | - | - | 86 | - | 0 | 0 | 14 | 71 | 57 | 14 | 43 | - | - | - | 0 | (Bazaid et al., 2022) |
|  | 18.4-19.7% among *KP* infections, 2.8-3.72% among uropathogens | Urine samples from UTI patients (in + outpatients) | 58.3-100 | 69 | 7 | 8.3-100 | - | - | 98-100 | 100 | - | 100 | 50-100 | 7 | 0 | 0 | 22-41.7 | 0-83.3 | 0-33.3 | 16.7-85 | 0-48 | - | 0 | - | 41.7 | (Bazaid et al., 2021, Alzahrani et al., 2020, Balkhi et al., 2018) |
|  | 5.9% of all urinary infections and 9.1% of *KP* causing urinary infections | Urine (ICU patients) | - | - | - | - | - | - | - | - | - | - | - | - | - | - | - | - | - | - | - | - | - | - | - | (Kabrah et al., 2021) |
|  | 0.81% of all uropathogens, 10% of *KP* causing UTI | Urine (female outpatients) | - | - | - | - | - | - | - | - | - | - | 0 | 0 | 0 | 0 | - | - | 0 | - | 0 | - | - | - | - | (Alasmary, 2021) |
| UAE | 20% among *KP* | Urine | 100 | - | - | - | - | - | - | - | - | - | - | - | 0 | - | - | - | - | - | - | - | - | - | - | (Ranjan Dash et al., 2018) |

Mixed source indicates all clinical specimens including lower respiratory tract infections, blood, urine, wound infections etc. AMP: Ampicillin, AMX/CLV: Amoxicillin/clavulanate, CX: Cefoxitin, CFT: Cefotaxime, CFZ: Ceftazidime, CFP: Cefepime, CFZO: Cefazolin, CFX: Cefixime; CXM: Cefuroxime, CTX: Ceftriaxone, ERP: Ertapenem, MNM: Meropenem, IMP: Imipenem, GEN: Gentamicin, AMK: Amikacin, TMPS: Trimethoprim‑sulfamethoxazole, FQ: Fluoroquinolone (Ciprofloxacin/Norfloxacin/Levofloxacin), NF: Nitrofurantoin, PIP/TAZ: Piperacillin/tazobactam, TG: Tigecycline, AZT: Aztreonam, COL: Colistin. - Not tested/not available. XDR: Extensively drug resistant, PDR: Pan drug resistant. *EC: E. coli,* EPEC: Enteropathogenic *EC,* EAEC: = Enteroaggregative *EC*, *KP*: *K. pneumoniae*, *EB*: *Enterobacteriaceae*, HAI: healthcare associated infection. *Drug not specified. - not reported.

**References:**

Abdallah, F. B., Lagha, R., Al-Sarhan, B., Mabrouk, I., Alhomrani, M., Gaber, A., et al. (2020). Molecular characterization of multidrug resistant e. coli associated to Q51 urinary tract infection in taif, Saudi Arabia. Pak J. Pharm. Sci. 33, 2759–2766.

Alfouzan, W., Dhar, R., Abdo, N. M., Alali, W. Q., and Rabaan, A. A. (2021). Epidemiology and microbiological profile of common healthcare associated infections among patients in the intensive care unit of a general hospital in Kuwait: a retrospective observational study. J. Epidemiol. Glob Health 11, 302–309. doi: 10.2991/ jegh.k.210524.001.

Al-Garni, S. M., Ghonaim, M. M., Ahmed, M. M. M., Al-Ghamdi, A. S., and Ganai, F. A. (2018). Risk factors and molecular features of extended-spectrum beta-lactamase producing bacteria at southwest of Saudi Arabia. Saudi Med. J. 39, 1186–1194. doi: 10.15537/smj.2018.12.23273.

Alzahrani, M. A., Sadoma, H. H. M., Mathew, S., Alghamdi, S., Malik, J. A., and Anwar, S. (2021). Retrospective analysis of antimicrobial susceptibility of uropathogens isolated from pediatric patients in tertiary hospital at Al-baha region, Saudi Arabia. Healthcare (Basel) 9. doi: 10.3390/healthcare9111564.

Ardati, K. O., Chacko, S. T., Jagtap, A., and Jacob, S. (2019). Prevalence of bacterial pathogens and antimicrobial susceptibility pattern in Bahrain tertiary care hospital. J. Bahrain Med. Soc. 31, 5–16. doi: 10.26715/jbms.31_06032019.

Badger-Emeka, L. I., Al-Sultan, A. A., Bohol, M. F. F., Al-Anazi, M. R., and AlQahtani, A. A. (2021). Genetic analysis, population structure, and characterisation of multidrug-resistant klebsiella pneumoniae from the Al-hofuf region of Saudi Arabia. Pathogens 10. doi: 10.3390/pathogens10091097.

Kabrah, A. (2022). Extended-spectrum beta-lactamase and carbapenem-resistant gram-negative pathogens in makkah, Saudi Arabia. Ethiop J. Health Sci. 32, 1221–1230. doi: 10.4314/ejhs.v32i6.20.

Lagha, R., Ben Abdallah, F., Aah, A. L., Amor, N., Hassan, M. M., Mabrouk, I., et al. (2021). Molecular characterization of multidrug resistant klebsiella pneumoniae clinical isolates recovered from king abdulaziz specialist hospital at taif city, Saudi Arabia. J. Infect. Public Health 14, 143–151. doi: 10.1016/j.jiph.2020.12.001.

Mutair, A. A., Alhumaid, S., Alawi, Z. A., Zaidi, A. R. Z., Alzahrani, A. J., Al-Tawfiq, J. A., et al. (2021). Five-year resistance trends in pathogens causing healthcareassociated infections at a multi-hospital healthcare system in Saudi arabi -2019. J. Glob Antimicrob. Resist. 25, 142–150. doi: 10.1016/j.jgar.2021.03.009.

Saleem, M., Syed Khaja, A. S., Hossain, A., Alenazi, F., Said, K. B., Moursi, S. A., et al. (2023). Pathogen burden among ICU patients in a tertiary care hospital in hail Saudi Arabia with particular reference to beta-lactamases profile. Infect. Drug Resist. 16, 769– 778. doi: 10.2147/IDR.S394777.

Shaaban, O. A., Mahmoud, N. A., Zeidan, A. A., Kumar, N., and Finan, A. C. (2021). Prevalence and resistance patterns of pediatric urinary tract infections in Bahrain. Cureus 13, e20859. doi: 10.7759/cureus.20859.
